# Hybridized Tungsten Oxide Nanostructures for Food Quality Assessment: Fabrication and Performance Evaluation

**DOI:** 10.1038/s41598-018-21605-5

**Published:** 2018-02-20

**Authors:** Pankaj Kumar, Prashant K. Sarswat, Michael L. Free

**Affiliations:** Department of Metallurgical Engineering, University of Utah, Salt Lake City, UT 84112 USA

## Abstract

Tungsten oxide based micro and nanosized structures possess good capacitance as well as enhanced rate capability. Such properties are useful in various applications including electrochemical supercapacitors. Apart from supercapacitance, WO_3_ and their 2D integrated structures have been modified using different methods to widen their range of the utility. Modification using layer coating, functionalization with other nanomaterial or molecules are methods that can be used to improve the core structure of WO_3_. But such modifications often alter electrochemical performance. The effects and outcomes of such modifications incorporated in WO_3_ structures were studied using electrochemical methods, sensing behavior, and morphological examination. One goal for such modifications was to improve robustness of the WO_3_ structures apart from any change in supercapacitance performance. After detailed electrochemical analyses of WO_3_ structures, a preliminary study was performed regarding the feasibility of the WO_3_ based sensors for food safety applications based on electrochemical detection of hazardous dyes in food. Preliminary results obtained after various electrochemical tests including pulsed voltammetry, cyclic voltammetry, and electrochemical impedance spectroscopy suggest the viability of WO_3_ structures for food safety applications.

## Introduction

Highly porous and thin electrochemical supercapacitors are proven ideal candidates for the variety of sensing applications due to superior thermal and chemical stability, large surface area, unique charge transport mechanism, and mechanically stability^1–3^. In general, due to the limitation of charge accumulation on the electrode, a limited specific capacitance and a low energy density are observed in electrostatic super capacitor (EDLS)^4,5^. Pseudo capacitors on the other hand, show a high specific capacitance as well as high energy density and also can be used for trace level sensing applications^4,5^. The Ultrathin layers of transition metal dichalcogenides (TMDCs) and their analogues have been researched recently among other chalcogenides due to enormous extension and variability in terms of applications and durability. Among the useful applications, their utility in electronics, sensing, and energy storage applications exhibited the revolutionary findings that can be linked with their performance. Hybrid electrical vehicles and digital communications need high power sources, and pseudocapacitors can fulfill these requirements^6^. The advantage of the supercapacitors is the utility of accumulated charges generated during faradaic reactions that result in higher capacitance than double layer capacitors^6^. Some of the representative examples are TaSe_2_, Nb-chalcogenides, HfS_2_, Mo-chalcogenides, W-chalcogenides, and V-chalcogenides^7^. It should be noted that pretreated chalcogenides extend their properties from other superconductors (as in the case of Ta-chalcogenides) to insulators (as in the case of HfS_2_). Electrochemical capacitors, often known as supercapacitors, can efficiently exhibit faster charging-discharging, long cycle life, high power density, and a simple method of energy storage. In these groups and their family, 2D materials and metal chalcogenides are often used for supercapacitor applications mainly due to large in-plane conductivity and enhanced surface area. Metal chalcogenides (e.g. some selected sulfides) are electrochemically active materials but their applications are limited. Large surface area, enhanced structural stability, conductivity, and an ease of fabrication are some prerequisites for an electrochemically active material. W-based sulfides/chalcogenides possess intriguing properties mainly due to layers of S-W-S that are covalently bonded but disjoined by a Van der Waals gap. Such an interaction is also responsible for keeping adjacent sulfur sheets intact.

Among oxides, many different transition metal based oxides have been utilized for pseudocapacitor applications, apart from selected conducting polymers^6^. Note that, not all transition metal based oxides are economically suitable for supercapacitor applications, especially Ru-oxides. Hence, other oxides were investigated for the supercapacitor applications. The poor rate performance and reduced electrical conductivity restrict the use of Mn-oxides for supercapacitor applications. Hence, a material that is more conductive with pore structures is more suitable. In this line of investigation, ordered mesoporous WO_x_ (tungsten oxide) was rigorously investigated. The WO_x_ possesses high electrical conductivity ~1.76 S cm^−1^ as well as large surface area ~54.3 m^2^ g^−1^, and hence fulfils the requirement of ‘an efficient supercapacitor electrode material^6^. Mesoporous WO_x_ exhibited a stable cycle performance ~1200 cycles as well as the stability in acidic electrolyte^6^. In order to reduce the capacitance losses at high scan rates, appropriate ionic/electronic conductivities are essential^8^. In a design point of view, the mesoporous electrode should be integrated on a metal current collector that will be durable for long term usage. Attempts have also been made to substantially improve the properties of the mesoporous material and the main focus was given to improve surface area, reduce the diffusion path length for ions, and provide facile access to the electrode. The direct growth of a mesoporous material on metallic substrates, especially nanowires or arrays can not only serve the preceding purpose but also provide mechanical robustness of the electrodes. The reduced capacitive loss is another addition in a series of these advantages.

In order to match the needs of lightweight portable electronic devices, sensors, and embedded supercapacitors, more electroactive materials along with tubular and hierarchical architectures are needed. The unique topography of hierarchical structures essentially promotes the ion diffusion and charge transport and thus enhanced cyclic stability^9^. Specifically, mesoporous WO_x_ was not only utilized for supercapacitor applications but also for a variety of sensing applications including gas sensing^10^, chemical sensors^11^, pH sensing^12^, and detection of pharmaceutical products^13^. Note that few bulk semiconductor materials show the measurable change in presence of very small amount of analytes (in ppm). However, the advantage of the nanostructured porous material is the rapid penetration as well as the removal of the analytes. Fortunately, nanostructured WO_3_ (enhanced specific surface area (SSA) as discussed in preceding section) works very well in this regards^14^. Another important characteristic of the semiconductor materials is change in the electrochemical behavior in presence of certain analytes. Few semiconductor materials show the measurable change in presence of very small amount of these analytes (in ppm). These characteristics of metal oxide lead to its use in sensor application. For example, TiO_2_ has been widely used for sensing organic compounds^15,16^. The sensing of the these analytes with the semiconductor is due to fact that in presence of the analytes, the electric dipole moment of the semiconductor materials changes when interact with the dipole moment of the analytes^14^. Also, it has been said that band gap of these semiconductor materials is important for the observable change^15^. For example, detection of electrochemical behavior for TiO_2_ due to narrow band gap (3.2 eV). Similar to TiO_2_, during the electrochemical degradation of organic compound, the electron generated can easily be detected in form of increase in electric current due of low band gap of WO_x_^17^. In a study^18^, application of the tungsten oxide based nanofilm for the detection of ethanol, NO_3_ and NO_2_ in gas phase up to 1000 ppm, 10 ppm and 3 ppm respectively, in a wide temperature range is reported. In the same line, ponzoni *et al*.^19^ showed the application of tungsten oxide for selective detection of NO_2_ to a level of 50 ppb. The detection limit of the tungsten oxide was further increased by surface modification using gold particles^20^. However, use of the nanostructured tungsten oxide is moreover limited to the gas phase sensor application^21–23^. Recently, food quality and associated safety have become very important to prevent foodborne illnesses. A possibility of food spoiling exists due to wrong practices related to improper food additives, colors, and labeling. The detection of these harmful chemicals have been using a sophisticated high performance liquid chromatography^24^. Although, the spectroscopy detection techniques are powerful analytical method and widely used, these techniques are time consuming, expensive, and require specific sample preparation. Electrochemical detection method on the other hand is rapid, cost effective and easy to operate. Recently, few electrode materials are developed for the electrochemical detection of the dye chemicals such as rhodamine^25–27^. For example, glassy carbon electrode shown to detect the rhodamine with a limit detection of 2.93 mg L^−1^ ^26^. In order to improve the reproducibility, Cu@carbon sphere electrode was developed but the sensitivity was limited^25^. cyclodextrin-functionalized nanogold/hollow carbon noospheres electrodes were recently developed to enhance the sensitivity of the detection^27^. However, most of these studies, are reporting only the carbon based electrode system for rhodamine detection. None of the studies have shown the metal oxides based electrode materials for the rhodamine detection. Even though these materials exhibited good performance, one concern regarding some of the carbon based electrode is dissolution of the electrodes in the case of excessive organic solvent based tests^28^. Hence, a search for the alternative electrode material is a must. Metal oxides are known to have outstanding sensing properties such as high selectivity and sensitivity, chemical and thermal stability, low cost, ease of synthesis, and ease of modification for sensing other analytes. Considering the advantages of the metal oxides for sensors applications an attempt is made to develop the WOx electrodes for the synthetic dye detection.

**Figure 1:**
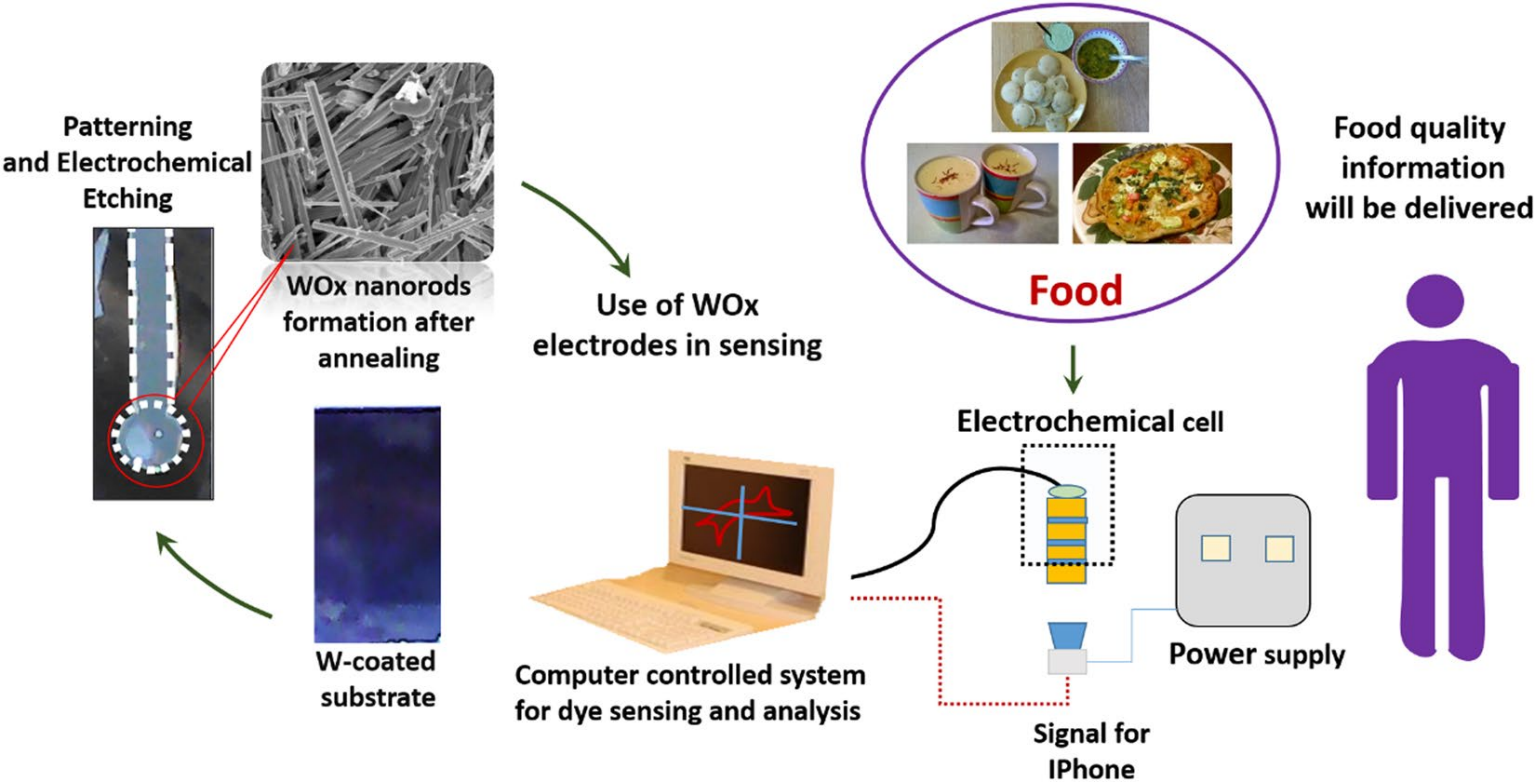
Fabrication steps for WO_x_ based electrochemical sensor and its use for food quality analysis. In a typical sensing unit WO_x_ electrode was used as a working electrode that was prepared using electrochemical etching and annealing steps.

In the present work, we investigated the possibility to enhance robustness of the nanostructured WO_x_ films by coating the film with the other metal chalcogenides. In addition, we explored the possible use of WO_x_ based sensors to address these concerns (See Fig. 1).

## Results

**Figure 2:**
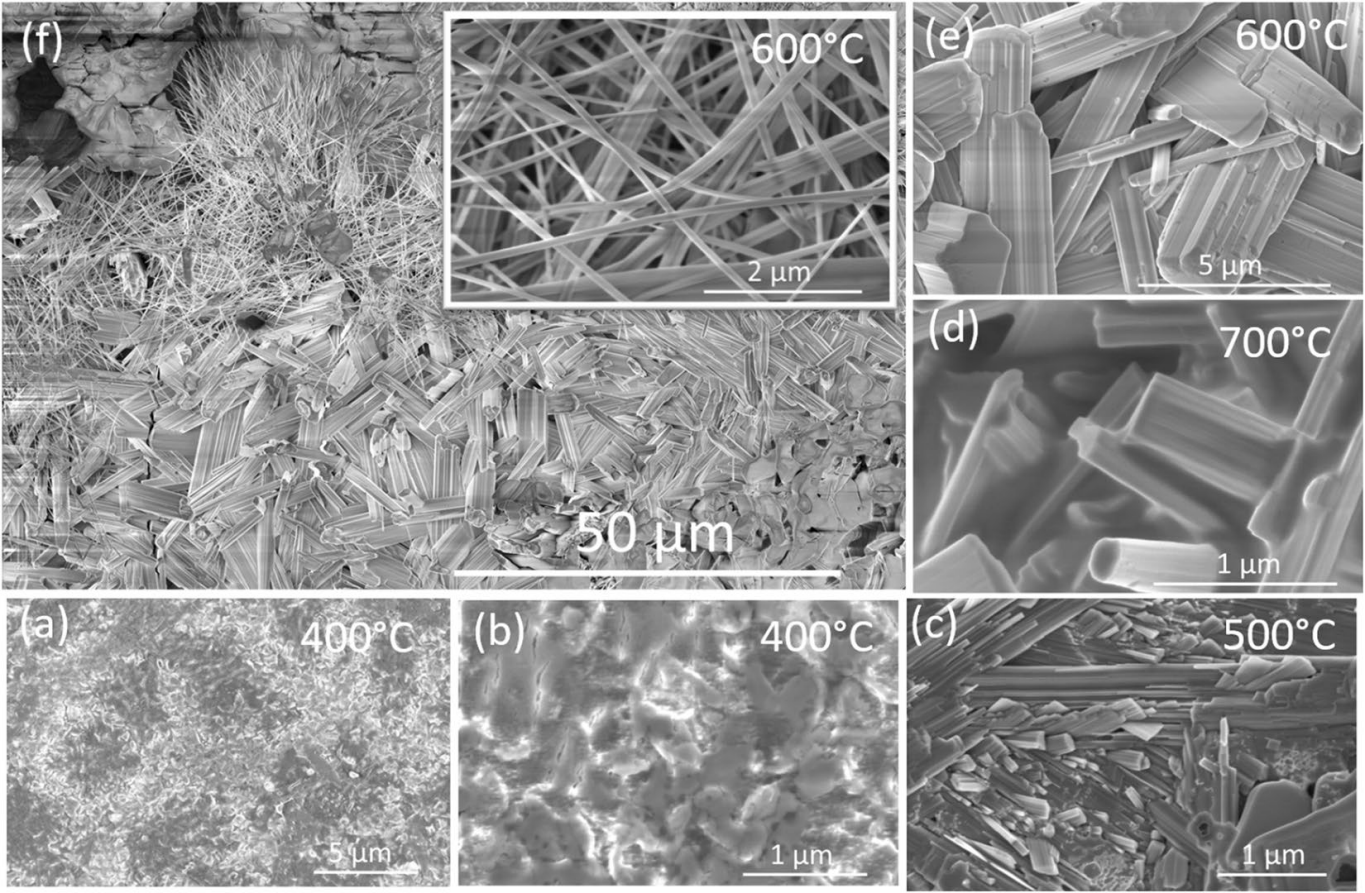
Scanning electron micrographs of WO_x_ layer annealed at various temperatures on tungsten coated Si substrate: (**a**) low magnification and (**b**) high magnification SEM image of WO_x_ layer annealed at 400 °C indicates the absence of WOx rods. (**c**) SEM image of WO_x_ layer annealed at 500 °C, indicates the incipient formation of WO_x_ rods. (**d**) SEM image of WO_x_ layer annealed at 700 °C, indicates that the WO_x_ rods start to dissolve at this temperature. (**e**) High magnification SEM micrographs of the WO_x_ layer annealed at 600 °C, indicate the formation of a fine rod morphology (**f**) SEM micrographs of the WO_x_ acquired at relatively lowers magnification to see global variation in the sizes and the shapes of the rods, inset shows the fines WO_x_ needles formation at this temperature.

The morphology of each deposited metal layer is crucial for pseudocapacitive performance. The morphology of WO_x_ layer on tungsten coated silicon obtained by annealing at different temperatures is shown in Fig. 2(a–f). At 400 °C, the WO_x_ rods do not form as evident from the Fig. 2(a,b). A lower magnification (Fig. 2a) SEM image of the sample, annealed for 400 °C, shows a flat granular morphology. At higher magnification (Fig. 2b) a discontinuous film is observed, however, there is no nano rod formation observed at this temperature. When the annealing temperature was increased to 500 °C, a significant change in the WO_x_ film was observed (Fig. 2c). The WO_x_ nano rods started to form at this temperature as shown in Fig. 2c. At 600 °C, the complete rod-like morphology of WO_x_ can be observed (Fig. 2(e,f)). The lower magnification SEM micrograph (Fig. 2f) of the WO_x_ layer formed by annealing at that temperature shows the distribution of WO_x_ rods on the tungsten coated silicon substrate. As can be seen, the WO_x_ rods of length up to ~10 µm and the diameter ranging from ~2 nm to 2 µm, have nano-grass morphology. The inset in Fig. 2f shows the high magnification image of the nano-grass morphology. The diameter of the grass varied from ~10 nm to ~100 nm. When the annealing temperature increased to 650 °C, the formation of WOx rods was more uniform. Also, no nano-grass type structure was observed at this temperature. Figure S1(a–c) shows the SEM micrographs of WO_x_ layer deposited by annealing at 650 °C on tungsten coated silicon substrate. Figure S1(b) indicates a uniform deposition of WO_x_ layer. The rod-like morphology of WO_x_ in the layer is evident from Fig. S1(b and c). The rod length varied from ~100 nm to ~10 µm while the diameter was less than ~5 µm at this temperature. The synthesis of KOH mediated micro-rods and associated formation mechanism has been discussed in earlier reports^9,29^. At 400 °C the size of rods was ~50–90 nm that subsequently increases with increase in temperature. It can be seen in our study that the size (diameter/width and length) of the tube extends from nanometers to a few microns, and that is consistent with their annealing temperature. It was observed that their length also increases with temperature. When the temperature increased to 700 °C, a partial dissolution of WO_x_ rods in the film was observed as shown in Fig. 2d. This indicates that at this temperature, the WO_3_ micro-rods were not stable, therefore, for further studies, the WO_x_ films grown at an annealing temperature of 650 °C were chosen for analysis and sensor fabrication.

**Figure 3:**
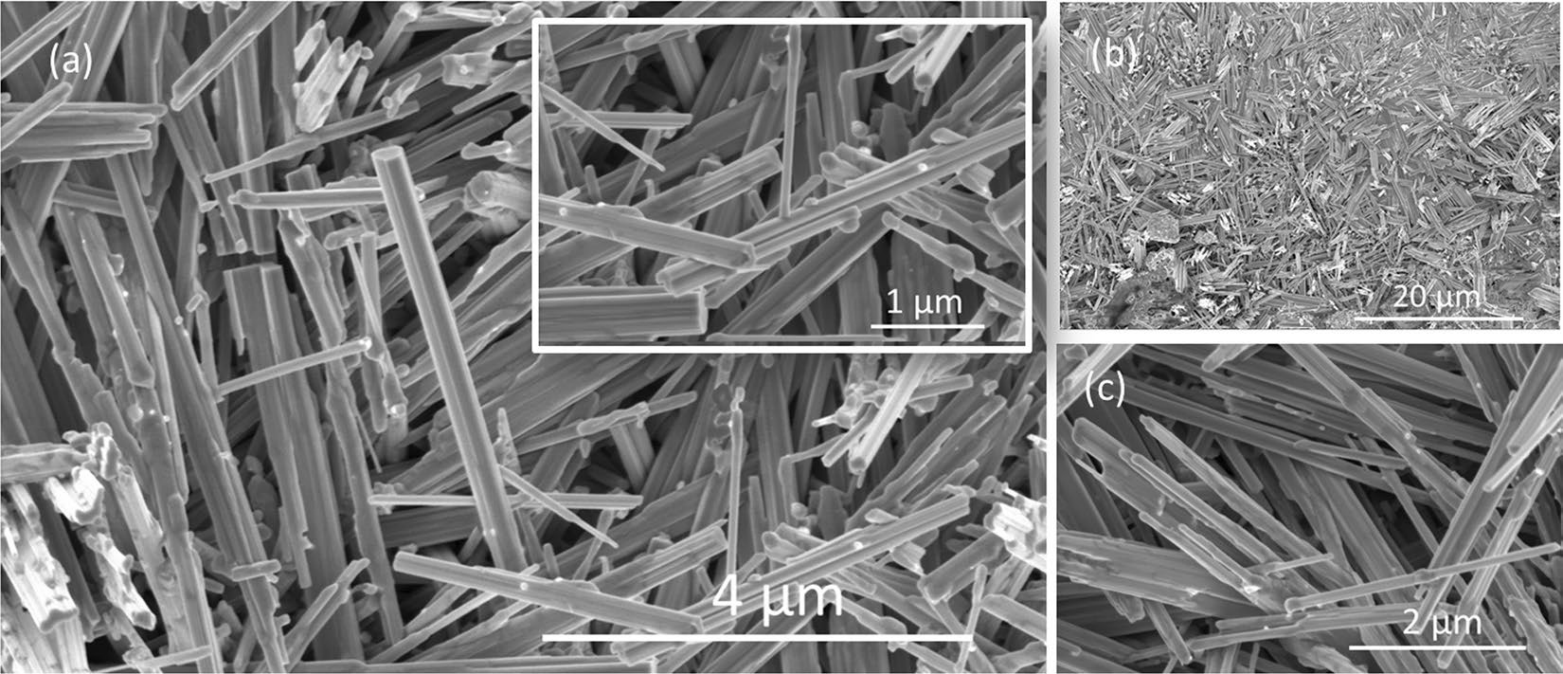
Scanning electron micrographs of WO_x_ layer on tungsten: (**a**) High magnification SEM image of WO_x_ layer, an inset shows the size and the shape of the rods indicates that the rods are finer and continuous compare to the WO_x_ rods grown on W coated Si (**b**) SEM image of the WO_x_ layer at lower magnification shows a relatively dense WO_x_ rods. (**c**) SEM micrographs of the WO_x_ acquired at relatively higher magnification shows relatively low variation in size and shapes.

The WO_x_ rods obtained by electrochemical etching and subsequent annealing at 650 °C of tungsten film show a relatively low variation in length compared to rods grown on Si substrate, as shown in Fig. 3. It was also observed that features on WO_x_ layer grown on tungsten is denser than on silicon substrate (Fig. S1b and Fig. 3b). In Fig. 3c, uniform WO_x_ rods in size and shape with the length 4 µm and diameter ~100 nm can be seen. A relatively low WO_x_ rod density is observed on the silicon substrate that may be due to the insufficient tungsten available for WO_x_ formation on silicon, since the tungsten thickness was less than 1 µm on the silicon substrate.

SEM micrographs in Fig. S2(a–c) show the surface morphology of CdS layer deposited on WO_x_. The deposited layer appears to be porous and adsorbed non-uniformly on the surface of WO_x_ microrods. The duration of deposition is also an important factor for film morphology, CBD was restricted (in this case) for shorter duration in order to avoid pore filling. The local growth of the CdS can be seen in the nanorods, and the coverage of the film on the WO_x_ increases with time. The high magnification SEM image (Fig. S2(b and d)) shows irregular rough shaped grains of CdS. The EDS mapping of the CdS layer provides the elemental distribution of Cd, S, W and O in Fig. S2(d–h).

The morphology of WSe_2_ film is shown in Fig. S3. In Fig. S3a, a rough uniform morphology of the film is observed. When the SEM micrographs were captured at high magnification (Fig. S3b), highly oriented grains were observed. The morphology looks like very thin flakes standing on the surface and matches well with earlier reported oriented features of WSe_2_^30,31^.

In Fig. S4, SEM images of Te coated WO_x_ substrates are shown. The low magnification SEM images (Fig. S4b) indicated the formation of an adherent and more uniform coating of tellurium (Te). A higher magnification image suggested the formation of a granular structure of Te. It can be observed that these granular structures are WO_x_ rods that are protruded out from a continuous film. Thus, it can be said that a thin continuous Te film is formed on a substrate. However, the thickness of the film is lower than the size of the WO_x_ rods as evident from Fig. 2a. In other words, the growth of Te follows a pour filling mechanism, rather than a non-uniform concentrated growth (see other cases discussed) on specific portions of WO_x_ rods. It also seems that Te growth occurred by filling the gap between the rods, and probably due to insufficient deposition time, the layer could not grow to a thickness that covers the size of the rods. The selected area for EDS mapping is shown in the inset of Fig. S4a. The elemental maps of the major constituents are also shown as insets of the Fig. S4a. The EDS maps show the uniform distribution of W, O, and Te. These results confirm the hypothesis of uniform deposition of Te layer on the substrate but in insufficient thickness.

Previous studies on Te layer deposition indicate that the morphology of the Te films is markedly affected by the substrate temperature and the deposition rate. Preferential growth of Te has been reported and the extent of preferential grain growth depends on the substrate temperature^32^. In the present study we do not see any preferential growth of Te film within the same temperature range. Therefore, it can be argued that the nature of the substrate in the present case, has a substantial effect on Te film growth. The previous study on Te growth indicates that the Te grows preferentially along certain directions on the substrate. The basic mechanism involves a preferential nucleation and growth of Te grains. In the present case, the WO_x_ substrate does not provide any preferential nucleation site because of its morphological constraints. The Te vapor deposits within the rod gaps and fills them. Therefore, the basic mechanism is different in the sense that the Te does not grow from preferential nucleation sites. The growth of Te in the present case, therefore, can be understood in terms of void filling which results in very strong and smooth adherent film.

**Figure 4:**
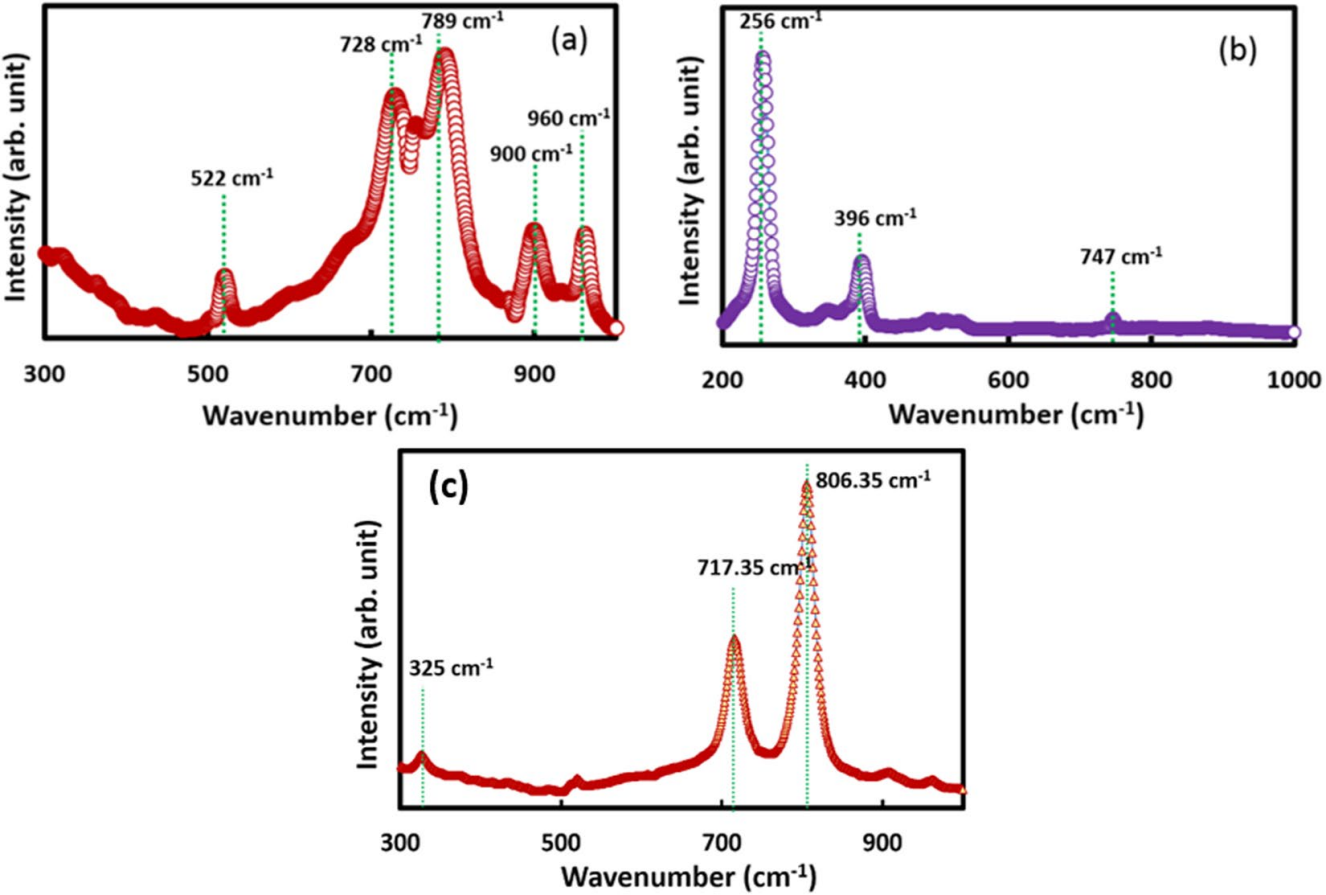
Raman spectra of (**a**) WO_x_ layer on tungsten shows the peaks at 522 cm^−1^, 728 cm^−1^, 789^−1^, 900 cm^−1^ and 960 cm^−1^ (**b**) Se layer on tungsten shown the peaks at 256 cm^−1^, 396 cm^−1^ and 747 cm^−1^ with the highest peak at 256 cm^−1^; (**c**) Raman sharp bands have been observed for WO_x_ grown on tungsten substrate (grown after electrochemical etching). One band at ~717 cm^−1^ corresponds to asymmetric vibration of O–W–O bond; another band at ~807 cm^−1^ represent symmetric stretching.

The Raman spectra for the WO_x_ and WSe_x_ at 785 nm laser excited at ~140 mW for the range of 300–900 cm^−1^ and 200–1000 cm^−1^ respectively are shown in Fig. 4(a) and (b). An additional Raman spectrum is shown for WO_x_ film grown using method 2 in Fig. 4(c). In the Raman spectrum of WO_x_ film, 5 peaks located at 522, 728, 789, 900, 960 cm^−1^ can be seen with the highest intensity peak at 789 cm^−1^. Although the peak positions are in good agreement with Raman spectrum of crystalline WO_3_, the comparative study^33^ indicates that this peak corresponds to different structural states of WO_3_. For example, 789 cm^−1^ corresponds close to orthorhombic β phase while peak at 728 cm^−1^ shows the presence of monoclinic γ-WO_3_. Thus, it can be argued that a mixture of different structural states of WO_3_ is present in the WO_3_ film at room temperature. This result supports the previous finding of a mixture of different phases of WO_3_ at room temperature^34^. However, it is believed that orthorhombic β phase is dominant in the film owing to its highest intensity in the Raman spectrum. It is to be noted that 960 cm^−1^ peak corresponds to nanocrystalline WO_3_^35^, which confirms the nanocrystalline formation of WO_3_ films on the tungsten substrate. The formation of 900 cm^−1^ peak may be related to a different organization of elemental WO_6_ octahedral^35^.

Our Raman spectrum measurement of WSe_x_ in the range of 200–1000 cm^−1^ reveals that the most prominent Raman peak for WSe_x_ film is located at 256 cm^−1^. The other dominant peak observed at 396 cm^−1^. These peaks are consistent with that of WSe_2_ Raman spectrum^36^. The Raman peak at 256 cm^−1^ is characterized by the formation of a bilayer film of WSe_2_^30^. Also, a peak located at 396 cm^−1^ corresponds to the WSe_2_ monolayer^36^. Therefore, the Raman analysis indicates bilayer deposition of the WSe_2_ film. This result coupled with the SEM micrographs indicates the formation of the flaky bilayer of WSe_2_ on WO_3_ substrate. We have also shown a Raman spectrum of WO_3_ grown on tungsten substrate (after electrochemical etching and annealing). It can be seen (see Fig. 4(c)) that this spectrum matches well earlier reported Raman spectra of crystalline monoclinic WO_3_^37^, but it is a little different from WO_3_ grown on silicon substrate.

**Figure 5:**
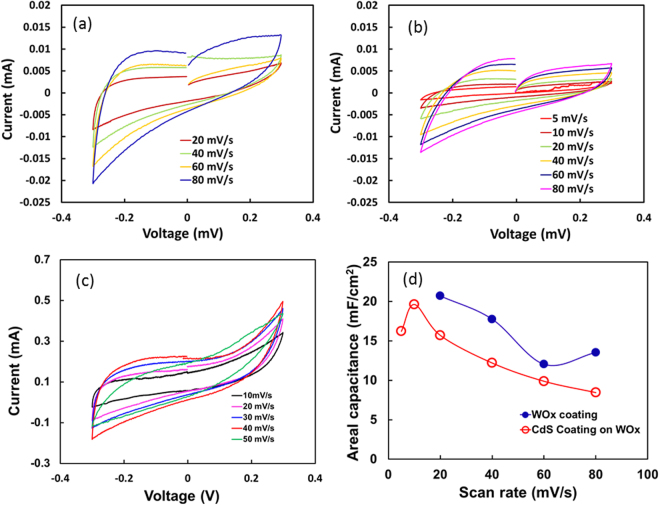
Electrochemical characterization: CV curves at various scan rates for (**a**) WO_3_ layer on W coated silicon (**b**) CdS later on WO_3_ layer (**c**) WO_3_ on tungsten substrate shows EDLC reaction over faradic adsorption (**d**) areal capacitance of WO_3_ and CdS coating on silicon substrate calculated based using Eq. (4).

**Figure 6:**
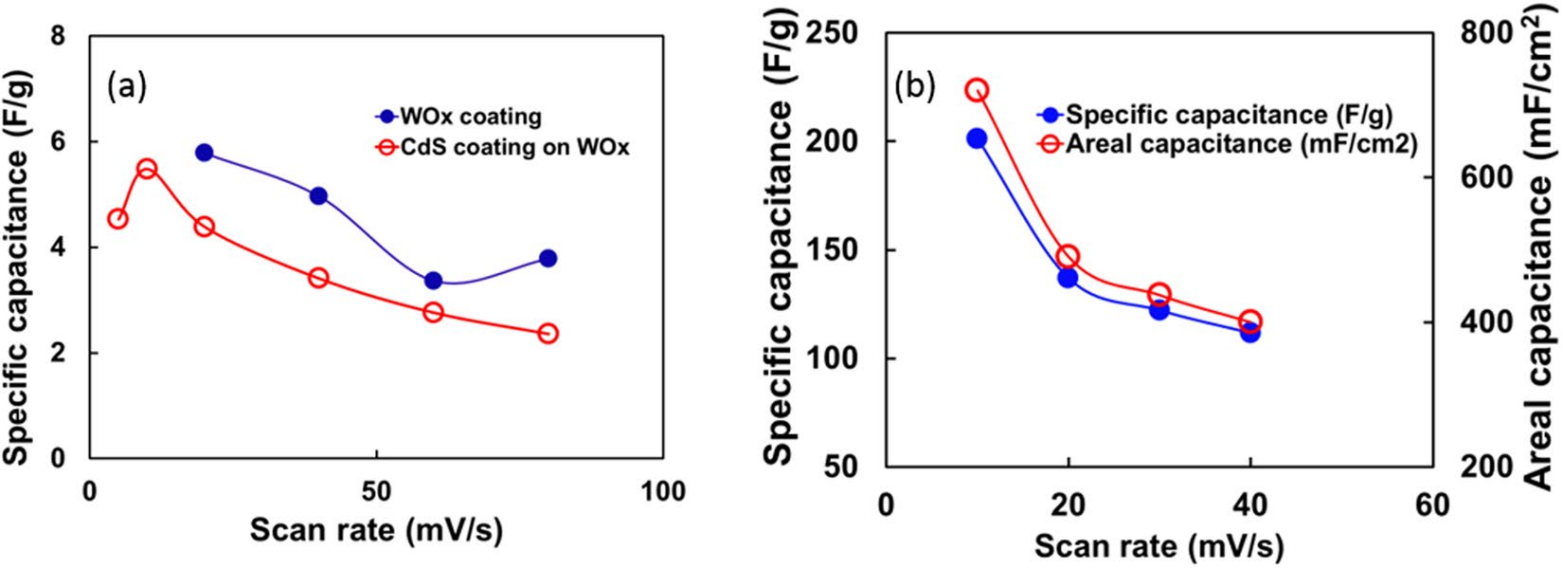
(**a**) The specific capacitance of WO_3_ and CdS coating on silicon substrate calculated based using Eq. (2) and (**b**) the specific capacitance and areal capacitance of WO_3_ on tungsten calculated using Eqs (4) and (5).

The typical cyclic voltammetry (CV) scans of WO_3_ and CdS coated WO_3_ on the silicon substrate, performed at various scan rates, ranging from 5 mV/s to 80 mV/s in voltage range of −0.3 V to 0.3 V are shown in Fig. 5a and b, respectively. Also, the CV curve for WO_3_ grown on tungsten substrate is shown in Fig. 5c. The CV curves exhibit near symmetric loops for all scan rates indicating their good electrochemical performance. As can be observed, the CV trends obtained for film grown on Si substrate (Fig. 5a and b) are different than the CV trend for WO_3_ on W film. This difference in CV behavior can be attributed to the morphology of the films on the substrate. The morphological differences lead to different outer defects resulting in an overall different double layer capacitance and the EDLC reaction. This results in variation in CV behavior of the films on the Si substrate and on the W film. It can be observed that the electrochemical performance of WO_3_ layer with micro-rod morphology on tungsten substrate shows the best electrochemical performance among all. The calculated areal capacitance (C_a_) of various samples including WO_3_, CdS coated WO_3_ on silicon, and WO_3_ on tungsten substrate based on their respective CV curves are shown in Figs 5 and 6. The C_a_ obtained for WO_3_ and CdS coated WO_3_ on silicon at 20 mV/s scan rate are 20.7 and 15.7 mF/cm^2^ respectively which is significantly lower than the reported areal capacitance for WO_3_ material^38–40^. In addition, a small reduction in areal capacitance (20.7 to 15.7 mF/cm^2^) was observed when the CdS coating was applied to WO_3_ coating. The WO_3_ materials also showed good rate capability (maintaining ~65% capacitance from 20 mV/s to 80 mV/s). When CdS coating was applied on WO_3_ coating, the rate capability of the film reduced and maintain only about ~54% of capacitance from 20 mV/s to 80 mV/s. The reduction in pseudo capacitor performance by applying CdS layer on WO_3_ coating on the silicon substrate may be associated with the masking effect on the chemical species diffusion reactions by random CdS layer.

The authors also conducted the ultrasonication test to show the robustness of the coated film on the substrate (see supporting information-Fig. S5). It can be observed that the WO_3_ film is easily peeled off from the Si substrate after the ultrasonication for 5 minutes. However, the CdS coated film was intact on the substrate after the same ultrasonication time. This shows the coating of WO_3_ film with other metal chalcogenides enhances the robustness of the WO_3_ film on the substrate.

## Discussion

The charge storage mechanism in WO_3_ material involves both the faradaic contribution (pseudocapacitance) and nonfaradaic contribution from the electric double layer^41^. However, the pseudocapacitance is a dominant mechanism responsible for the total capacitance of the material. It has also been shown that pseudocapacitive charge transfer is a surface limited diffusive process^42^. Therefore, reduction in the areal capacitance in CdS coated WO_3_ layer can be attributed to the surface diffusion barrier produced by CdS layer.

The WO_3_ layer on tungsten electrode shows excellent areal capacitance (C_a_) of ~720 mF/cm^2^ at a scan rate of 10 mV/s which may be attributed to the large surface area of the WO_3_ micro rods. Most of the studies showing the evaluation of the pseudocapacity of WO_3_, however, adopt gravimetric capacitance as standard. In the present study, we have calculated both the areal (C_a_) and the gravimetric specific capacitance (C_g_) of WO_3_ films to compare with other studies. For gravimetric capacitance, the weight of the electrode materials was estimated by the thickness measurement of the WO_3_ layer on the substrate. A few studies^6,8,43,44^ reporting the areal capacitance of WO_3_ or hybrid WO_3_ materials 0.2 mF/cm^2^ (scan rate of 1 mV/s) to 684 mF/cm^2^ (scan rate of 10 mV/s). An aerial capacitance of ~2 mF/cm^2^ for porous WO_3_-carbon composites was reported by Jo *et al*.^43^. It is known that the large surface area of the electrodes possesses enhanced areal capacitance due to increased charge storage on the surface^8^. An aerial capacitance of value ~684 mF/cm^2^ was reported for nanoflower WO_3_ based electrode^41^. A high value of areal capacitance achieved in his studied of WO_3_ materials is attributed to the WO_3_ structure with large surface area. In the present study, the observed capacitance value (~720 mF/cm^2^ at 10 mV/s) of WO_3_ materials is close to the reported capacitance by Qiu *et al*.^41^. Thus, the large capacitance in the present study can be attributed to the large surface area of nano rods of WO_3_. The areal capacitance studies of nano rods of WO_3_ are rare. However, few studies report the specific capacitance of nano rods structured WO_3_ material. Pal *et al*.^45^ studied the capacitance of this material and reported a specific capacitance of ~482 F/g.

The specific capacitance of the WO_3_ material is varied from as low as 81 F/g to as high as ~800 F/g^38–40^. In this study, we observe a large variation in specific capacitance of the WO_3_ depending on the substrate. For the WO_3_ material coated on Si substrate shows Cg as small as 6 F/g at 20 mV/s (Fig. 6a). Even, CdS coating on WO_3_ seems no effect on the capacitance behavior and showed similar Cg as ~4 F/g at the same scan rate. When the WO_3_ was grown on the tungsten substrate, the Cg increased to ~200 F/g (Fig. 6b). An increased specific capacitance of WO_3_ on tungsten can be attributed to a relatively lower amount of WO_3_ on Si substrate due to an insufficient amount of tungsten for WO_3_ growth. The present Cg value of ~200 F/g is equivalent to the nano flower WO_3_ (196 F/g) reported by Qiu *et al*.^41^. Therefore, it can be argued that the large surface area obtained by growing nano rods is equivalent or better than the nano flower WO_3_ supercapacitance performance. However, the nano-rod structure is likely to facilitate charge conduction for charging and discharging more easily than nano flower due to the reduced number of contact points.

Although the specific capacitance (C_g_) is a more common approach to show the capacitance behavior of the materials, it is said that when the active electrode materials is small such as in micro devices the areal capacitance (C_a_) is more relevant^46^. In the present study, we showed the high areal capacitance achieved for WO_3_ and relatively high specific capacitance (C_g_) by increasing the surface area morphology.

The nanostructured morphology of WO_3_ showed an excellent balance of C_a_ and C_g_ compared to other reported values^38–40^. It is observed that the robustness of the nanorods morphology of WO_3_ can be further improved using layer structure and at the same time the electrochemical performance of WO_3_ can be optimized by layering with other functional materials. This study showed the potential application of nanorod-WO_3_ as a robust electrode material for energy storage devices.

Recently, one-dimensional nanostructured semiconductor metal oxides showed the potential for gas sensing application. This is due to the fact that these metal oxide structure provides the high surface to volume ratio and at the same time maintaining good chemical and thermal stability. The use of WO_3_ in sensor application has been shown recently. Its use as a selective and ultrasensitive sensor for the detection of NO and NO_2_ is shown^47^. WO_3_ has been used as potential pH sensor [9]. Due to its versatile sensor application, the nanostructured WO_3_ can also be utilized as sensor application for organic compounds. In the present study, the detection of organic compounds used in food processing is explored using nanostructured WO_3_ as sensing material. Given that food safety is a critical issue, the development of WO_3_ based sensor can provide an economic opportunity to use as the point-of-use sensor to establish the food screening and safety. In the following section, the experimental investigation of electrochemical sensing of organic compounds as methyl red, β carotene and Rhodamine 6G using the nanorod structured WO_3_ is discussed.

### Utility of WO_x_ for dye detection and food quality analysis

Synthetic edible chemicals are widely used in food industries to improve the flavor, taste, and appearance. A strict guideline must be followed while choosing these chemicals due to their use in edible foods^48^. A recent report indicated the use of synthetic non-edible colors that were used for food coloring^49^. Many of these chemicals are toxic and have the carcinogenic effect. One representative example is Rhodamine that is a potentially carcinogenic coloring agent that is found in food in some underdeveloped countries^50,51^. Due to its negative impact on human health, its use in food industries is controlled or banned in many countries. However, its illegal use is predominant in many food industries. Importing food from many countries introduces the risk of consuming these harmful chemicals. For example, high levels Rhodamine 6G were found in the imported vegetable and dairy products in the U.K^52^. Another example is Methyl Red, which was also found in some food products and has similar health issues.

It is important to note that the U.S. imports a large quantity of food from other countries. Some of these countries were major suppliers of food and agricultural products (~150,000 mt) for the U.S. in 2014^53,54^. For safety assessment, it is essential to establish a procedure to screen the contamination in imported food supply. Several analytical methods were developed and have been used in identifying the chemicals in food^55^. However, the associated complexity and cost with the current method for detection of these chemicals potentially restrict its use as a screening tool for the large quantity of food. Use of point-of-care sensors, therefore, can be utilized to screen this chemical in the food in bulk. In order to fulfill this requirement, we propose and demonstrate the use of nanocrystalline tungsten oxide in the detection of chemicals in contaminated food. Note that tungsten oxide was already used for detection of nitrogen oxide^47^, ammonia^18^, hydrogen sulfide^21^ and hydrocarbons^56^. In the present investigation, we have systematically studied the trace level sensing of selected edible and non-edible dyes using electrochemical tests with a fixed scan rate with nanocrystalline WO_3_ used in the working electrode. Although, it has been reported that a high scan rate was found to be suitable for improved sensitivity^57,58^, the present investigation focused on the utility of nanostructured WO_x_ for the detection of dyes.

The approach to this problem was to examine the cyclic voltammograms of different edible and non-edible dyes and document the differences in their responses. We have chosen edible dye (β carotene), and two non-edible dyes (Rhodamine and methyl red) for analysis.

### Analysis of cyclic voltammetry results for β carotene

**Figure 7:**
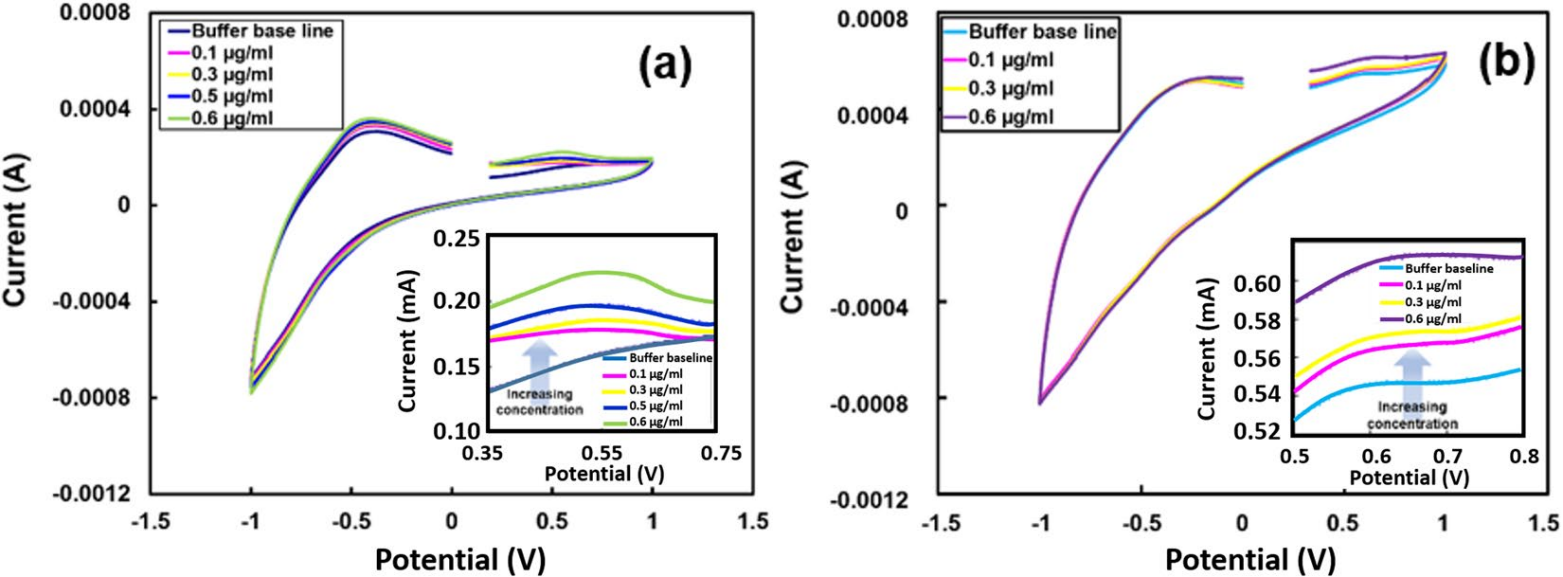
The cyclic voltammograms of phosphate buffer (base line) and buffer of different concentrations of (**a**) β carotene showing oxidation peak at ~0.6 V in the scan range of −1.5 V and 1.5 V. A local section of cyclic voltammogram in the range of 0.35 and 0.75 showing a very systematic change in peak current at ~0.6 V. An increase in peak current when β carotene concentration was increased from 0.0 µg/ml to 0.6 µg/ml in the phosphate buffer (**b**) methyl red showing peak at ~0.55 V in the scan range of −1.5 V and 1.5 V. A voltammogram section in the range of 0. 5 and 0.8 (inset) showing a systematic shift from ~0.55 V to ~0.65 V. Simultaneously increase in the peak current change can also be observed when methyl red concentration was increased from 0.0 µg/ml to 0.4 µg/ml in the phosphate buffer.

β carotene has been often used to provide yellow to orange color to food. Cyclic voltammograms using the three-electrode system cell with phosphate buffer and buffer with the different concentration of β carotene is shown in Fig. 7a. In the electrochemical cell, the nanorods of tungsten dioxide on tungsten substrate was the working electrode while a platinum plate was the counter electrode. A saturated calomel electrode was used as a reference electrode for the CV analysis. It can be seen that phosphate buffer shows a distinct peak at ~0.6 V in the scan range of −1.5 V to 1.5 V. A systematic shift of the peaks towards high current was observed when the β carotene concentration was increased from 0.0 µg/ml to 0.6 µg/ml. The peak shift is evident in the magnified portion of the graphs as shown in Fig. 7 inset. The observed change in current due to an addition of β carotene suggest that the nanostructured tungsten oxide has a potential for sensing β carotene in food.

### Analysis of cyclic voltammetry results for methyl red

Nanostructured tungsten oxide was also utilized in CV experiments for methyl red detection. CVs were recorded for methyl red in phosphate buffer solution of concentration from 0.0 µg/ml to 8.0 µg/ml. Figure 7b shows the cyclic voltammograms for methyl red buffer solution. An oxidation peak for the methyl red buffer solution with 0.0 µg/ml was observed at ~0.55 V (anodic). The peak at ~0.55 V shifts towards positive voltage from ~0.55 V to ~0.65 V when methyl red concentration increased from 0.0 µg/ml to 0.6 µg/ml. In addition to the peak shift, an increase in peak height can also be observed with the increased concentration of methyl red in buffer solution (inset Fig. 7). From these results, it can be concluded that tungsten oxide can be utilized for trace level detection of the methyl red using electrochemical techniques.

### Electrochemical impedance analysis for Rhodamine 6G

**Figure 8:**
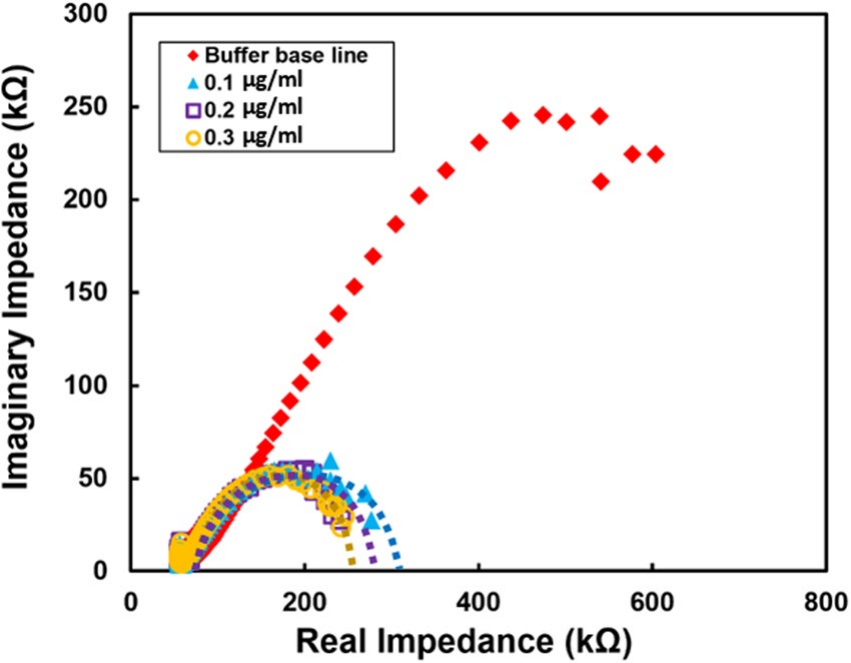
Nyquist plot of phosphate buffer (baseline) and the buffer of different concentrations of Rhodamine 6G, showing the reduction in diameter of the semi-circular region with the increase in concentration.

Electrochemical Impedance Spectroscopy (EIS) technique has inherent potential of detecting the chemical and biological change and has been reported to be effective in the fat content determination in meat^59^ as well as for the detection of several toxins in food^60^. This technique in based on determining the impedance (Z) of the system as a function of signal perturbance frequency. For present EIS experiment, a constant sinusoidal voltage was applied to the tungsten oxide sensor with a frequency sweep of 1 Hz to 10 kHz. To observe the sensor response due to the Rhodamine 6G, the phosphate solution was spiked with different concentrations of Rhodamine 6G varied from 0.0 µg/ml to 0.3 µg/ml. The Nyquist plot obtained for the different concentrations of Rhodamine 6G is shown in Fig. 8. The plot shows the semi-circular region with different diameters corresponding to Rhodamine 6G concentration in the test solution. In general, the Nyquist plots are analyzed based on Randel’s cell model concept^61^, where solution resistance, double layer capacitance, and the charge transfer is considered. In such model, double layer capacitance is parallelly connected with charge transfer resistance (some time it is called polarization resistance). Such a parallel connection system is again connected in series with solution resistance. However, one must note that this is a most simplified cell based model. In case of mixed kinetic and diffusion process, the analysis of model is more rigorous. In case of present WO_3_ morphology, because it is a slightly porous material, pore resistance of ion conducting channels will also come in the picture. In a realistic sense, for appropriate and detailed analysis, double layer capacitance, intact capacitance, as well as charge transfer should be considered apart from solution resistance and pore resistance. However, it is very clear that overall impedance of the system reduces due to addition of Rhodamine 6G. Hence it is most likely that oxidation causing the release of the electrons that in turn alters the charge transfer that can affect the EIS output as revealed in Fig. 8. The Rhodamine 6G molecules adsorbed on the tungsten oxide electrode surface as a result of electron transfer from the electrode. Therefore, the higher the concentration of the Rhodamine 6G, higher the charge transfer occurs leading to the relatively smaller diameter of the semi-circular region in Nyquist plot. This clearly indicates that semiconductor tungsten oxide can be utilized as the sensor for the detection of harmful Rhodamine in food.

### Sensing Mechanism

It has been reported^15,62,63^ that sensing capabilities of sensors are based on oxide conductivity adjustments that occur on the surface or near the surface of oxide based sensors as a consequence of electrochemical reaction with the organic molecules. The organic dyes, β carotene and methyl red, electrochemically oxidized and hence, can be corroborated with the oxidation peaks observed in the respective CV analysis (Fig. 7). The dye oxidation could be possible by electro-oxidation and the hydroxyl ions (OH^−1^)^64,65^. The adsorption of dyes and by-product formation due at the anode surface play an important role. It is well known that WO_3_ is an n-type semiconductor and in the presence of OH^−^ groups the conductivity of n-type sensors increases. Because of the presence of OH^−^ on the WO_3_ sensor surface, the formation of a stable complex with dye is possible, and such new complex electrochemical signature is different.

Studies have demonstrated the direct electro-oxidation of methyl red using anodes such as PbO_2_^65^ and TiO_2_^66,67^. These anodes at the surface generates hydroxyl radicals from the water as

1 $${{\rm{H}}}_{2}{\rm{O}}\to {}^{{\rm{\bullet }}}{\rm{O}}{\rm{H}}+{{\rm{H}}}^{+}+{{\rm{e}}}^{-}$$

The hydroxyl radicals oxidize the organic dyes. One of the early work has shown the complete oxidation of methyl red to CO_2_ by hydroxyl radicals generated by utilizing PbO_2_ as anode following the reaction^65^:

2 $${{\rm{C}}}_{15}{{\rm{H}}}_{15}{{\rm{N}}}_{3}{{\rm{O}}}_{2}+86{}^{\bullet }{\rm{O}}{\rm{H}}\to 15\,{{\rm{CO}}}_{2}+3\,{{{\rm{NO}}}_{3}}^{-}+3{{\rm{H}}}^{+}+49{{\rm{H}}}_{2}{\rm{O}}$$

The methyl red oxidation is facilitated following the two mechanisms. In the first mechanism, the hydroxyl ions generated by the oxidation of OH- or H_2_O by the photo-generated holes^68^ which oxidize the methyl group of methyl red. Another mechanism is based on the formation of hydroxyl radicals that react with the aromatic rings of the dye leading to the formation of hydroxylate by- product. The mechanism is known to be operative independently. In both the reactions, the creation of the hydroxyl radical is of great importance. In the present case where the photoelectron is absent, the hydroxyl radical is generated following Eq. 1. It is hypothesized that methyl red oxidation occurred due to the reaction of radicals with the aromatic rings which may lead to the formation of 3.3′ isomers compound^67^.

Similarly, electrochemical reaction causes β carotene to oxidize which leads to electron transfer. The electrochemical oxidation of β carotene study using a platinum electrode indicated that the irreversible oxidation process. The process of oxidation takes place by two electron transfer and the oxygen radical plays an important role. It is known that the oxygen-related defect sites of metal oxide are the most favorable for the adsorption of the organic molecules. Oxygen molecules can be dissociated and adsorbed at the oxygen vacant sites of the metal oxide. The organic β carotene molecule at these sites at the WO_3_ surface react with the oxygen molecules and generates the electron. The current signal that has been reported during this experiment may be a result of this reaction mechanism.

Similar mechanisms have been envisaged for the interaction of Rhodamine 6 G with the WO_3_ sensor. The oxidation species are adsorbed to the WO_3_ nanorod surface and react with adsorbed hydroxyl groups releasing water and a free electron following the reaction:

3 $${{\rm{OH}}}^{-}({\rm{ads}})+{{\rm{OH}}}^{-}({\rm{ads}})\to {{\rm{H}}}_{2}{\rm{O}}+{\rm{O}}({\rm{ads}})+{{\rm{e}}}^{-}$$

The adsorbed molecules can also be directly oxidized or dehydrogenated by the surface-adsorbed oxygen releasing free electrons to the oxide surface that effectively reduces the sensor resistance of the n-type WO_3_ nanorods as observed in Fig. 8. The increase in current response (oxidation peak height) with the increase in the concentration indicates the strong interaction between the sensor surface and the dye. The unique characteristics of WO_3_ nanorods in electron transfer and OH^−^ adsorption make it a suitable and highly favorable reaction. The structure of these nanorods provides not only stability and increased surface area of the interaction between the metal ions and organic dye but the rod structure also enables greater electron transfer during the oxidative interaction with minimal losses. The current signal that has been reported during this experiment may be a result of this formation of OH^−^ and the complex formation.

### Application of nanostructured tungsten oxide for the detection of the model dyes in food

**Figure 9:**
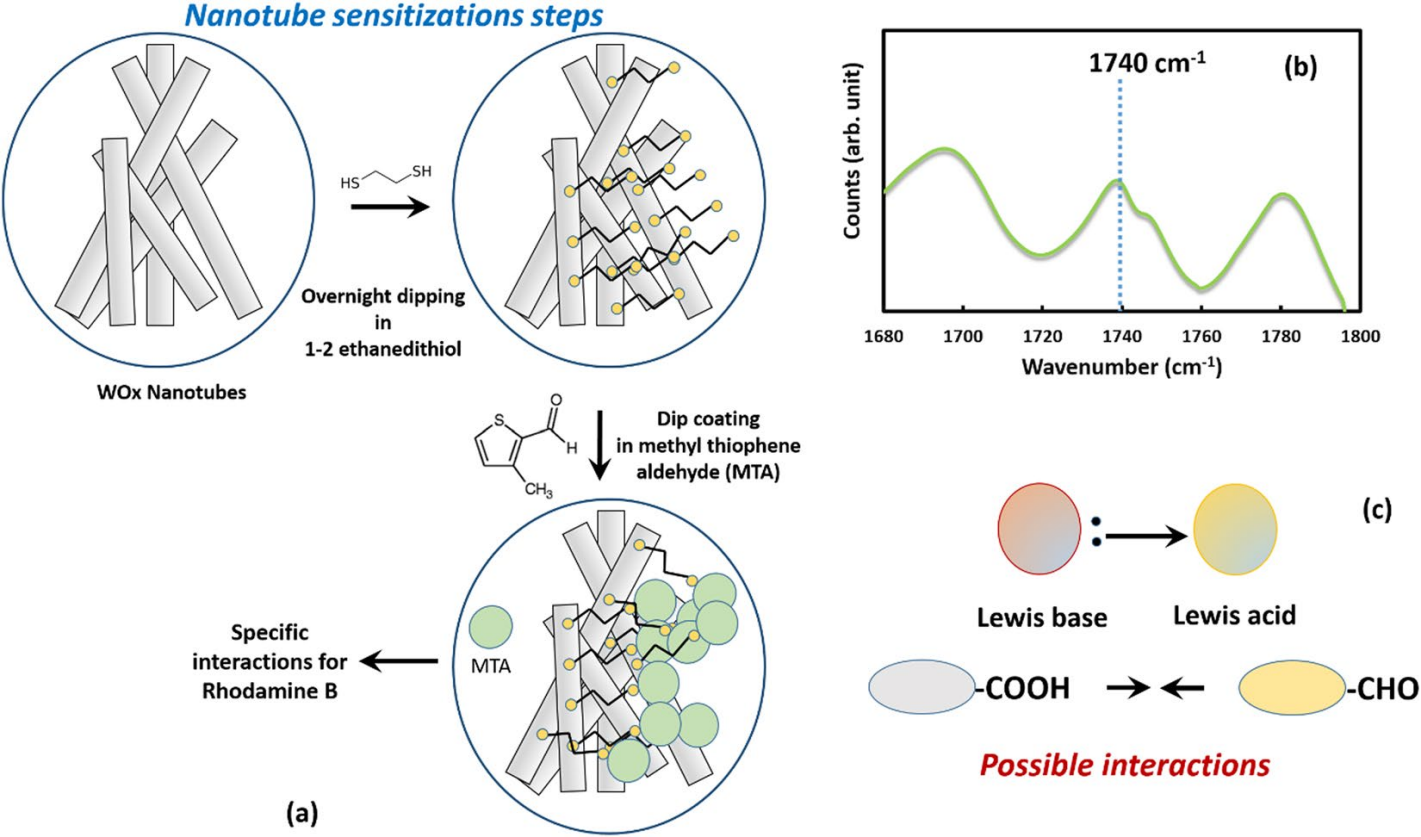
(**a**) A schematic diagram showing the surface modification (sensitization steps) on WO_x_ nanostructure with methyl thiophene aldehyde for selective detection of the organic dye molecule. The nanostructured rods of WO_x_ were dipped into the 1,2 ethanedithiol followed by dipping in the methyl thiophene aldehyde (**b**) Raman spectrum of the methyl thiophene aldehyde coating on nanotubular structure showing a characteristic peak at ~1740 cm^−1^ (for –CHO group); (**c**) possible interactions of analyte with sensing molecules.

It is demonstrated that the developed nanostructured tungsten oxide can detect the model dye color used in food processing in a concentration level as low as 0.1 µg/ml. In the present study, the detection tests of the model dyes (rhodamine 6 G and methyl red) in the real food sample have been performed using the surface modified (see Fig. 9 and method section) nanostructured WO_x_. Many different compounds can be proposed to sense Rhodamine 6 G, however, it should be noted that rhodamine 6 G contains carboxylic, ether, and amino group. The presence of variety of groups in single organic compounds makes it more complicate and challenging to detect the concentration accurately. As described in the previous section, the chemical species are adsorbed to the WO_3_ nanorod surface and react electrochemically with the reactant groups leading to generation of free electrons. Therefore, for a selective detection of the species there should be a good interaction of the organic species on the sensor. In order to achieve the selective adsorption of organic molecule we functionalized the WO_3_ nanostructured with methyl thiophene aldehyde. A recent report based on rotational spectroscopy concepts, suggests that carboxylic acid and aldehyde group can be bonded through classical OH-O and relatively weak CH-O bond^69^. The report also suggests that such interaction is quite rigid^69^. Therefore, it is expected that the functionalization leads to a bond formation between the nanostructured material and the chemical species. It is necessary to create an active group that is necessary for binding of the chemical species. Therefore, depending on the active group the chemical functionalization is required for better sensor performance. In this case, due to potential for steric hindrance of the amino group^70^ we restricted our study for detection of carboxylic acid group of rhodamine 6 G. Based on CV experiment, we show that Rhodamine 6 G in the concentration level as low as 2 ppm can be selectively detected using the surface modified WOx (Fig. S7). Apart from this interaction, Lewis acid-Lewis base interaction (See Fig. 9) is also very useful in many cases and improved the performance and selectivity of the sensor^71,72^. In our case of rhodamine 6 G, the use of aldehyde compound coating for WO_3_ surface functionalization serves these two purposes. It is important to note that the behavior of functional material coated electrode is unique because in this case the results are generally rationalized in terms of the electrostatic interactions between the dissolved species and the functional molecule coated on the electrode, apart from the occurrences of regular oxidation-reduction reactions^73–75^. Such an interaction can be attraction or repulsion, depending on the species and solution condition (pH, temperature, range of potential). Other advantages of these functionalization are enhancing the utility in the continuous flow system, protection from surface poisoning, modified electrocatalytic activity, and specificity towards analytes. The mixture of the phenol and 4-hydroxybenzaldehyde have been earlier utilized for serving this purpose. Using this type of method, a variety of compounds including dopamine (DA), glucose, and formaldehyde have been detected. Many different reports followed a similar model to explain sensing. Some examples are listed in references^73–75^.

**Figure 10:**
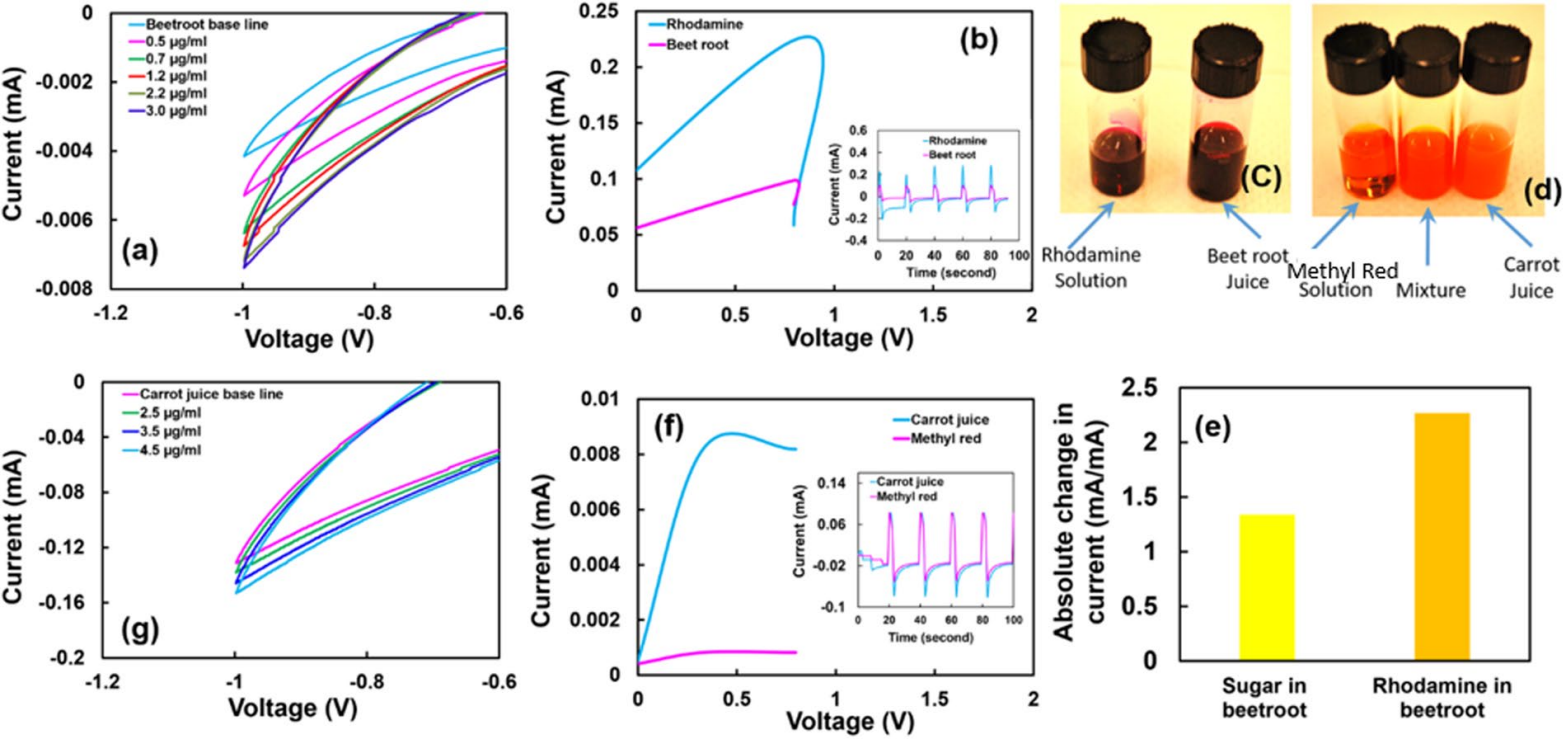
(**a**) A local cyclic voltammogram in the range of −1.2 to −0.6 V of beetroot juice (base line) and juice of different concentrations of Rhodamine 6 G showing a systematic increase in the current when the concentration was increased from 0.0 µg/ml to 3.0 µg/ml. An increment in current from 0.004 mA for 0.5 µg/ml to 0.007 mA for 3.0 µg/ml is evident. (**b**) The square wave voltammogram (SQV) of Rhodamine 6G and Beetroot showing a current response difference. A high current response was observed for Rhodamine 6G compare to the beetroot juice. Inset figure indicates the repeatability of sensor response from Rhodamine 6G and the beetroot juice. (**c**) Optical image indicates that visually no difference between the Rhodamine 6G solution and the beetroot juice. (**d**) Optical image showing no visual difference between Methyl red and the carrot juice. (**e**) Bar graph showing the absolute current response of the sensor when exposed to beetroot spiked with sugar (0.25 gm was added in 1 ml of beetroot juice) and beetroot with Rhodamine 6 G (4.5 µg Rhodamine in 1 ml beetroot juice). A significant higher current observed for rhodamine 6 G compared to the sugar. This clearly indicates the WO_3_ is selective towards the rhodamine in the food sample. (**f**) SQV of Methyl red and carrot juice showing the difference in current response. A high current response was observed for carrot juice compare to the methyl red solution. Inset figure indicates the sensor response is repeatable from Methyl red and carrot juice. (**g**) A local cyclic voltammogram section (−1.2 to −0.6 V) of carrot juice (base line) and juice of different concentrations of methyl red showing a systematic change in the current with the increased concentration. An increase in the current can be observed when methyl red concentration was increased from 0.0 µg/ml to 4.5 µg/ml in the carrot juice.

Beetroot juice and carrot juice were chosen as food samples since both produce the representative color of methyl red and rhodamine 6 G respectively (Fig. 10c and d). Figure 10(a and g) shows the partial cyclic voltammograms in voltage range of (−1.2 to −0.6 V) for Rhodamine 6 G and methyl red in beetroot juice and carrot juice of concentration from 0.0 µg/ml to 3.0 µg/ml and 0.0 µg/ml to 4.5 µg/ml respectively.

A systematic increase in the current response was observed when the concentration of the Rhodamine 6 G was increased in the beetroot juice (Fig. 10a). The current increased from 0.004 mA to 0.007 mA when the concentration increased from 0.0 µg/ml to 3.0 µg/ml respectively. To understand the response from the Rhodamine 6 G, square wave voltammetry (SWV) was used. As shown in Fig. 10b, a higher current response (~3 times) was observed for Rhodamine 6 G (higher peak current) compared to the current response from beetroot juice (relatively lower peak current). Also, when exposed to rhodamine 6 G and the beetroot for a given time period, there is a significant difference in the current response. The difference in current response is repetitive as shown in inset in Fig. 10b. These results clearly indicate that surface modified WO_3_ is very selective to the rhodamine 6 G. The authors also conducted square wave pulse test to compare the performance of the sensor for sugar addition in the beet root juice (see supporting information-Fig. S6). It can be seen that the current change was much less than in the rhodamine-beet root case. The current response observed from both the rhodamine and the sugar sample in beetroot juice is shown in Fig. 10e. As can be observed that the normalized current response for rhodamine 6 G contained beetroot is significantly higher (almost double) than the beetroot containing sugar even though the concentration of Rhodamine is 3 order of magnitude less than sugar in beetroot juice. This result clearly suggests that the surface modified nanostructured WO_3_ is very selective in detection of rhodamine in the food sample. The authors also conducted square wave pulse test to compare the performance of the sensor for sugar addition in the beet root juice (see supporting information-Fig. S6). It can be seen that the current change was much less than rhodamine-beet root case.

Similar to the rhodamine 6G, the current response increased with the increment in the concentration of methyl red in the carrot juice (Fig. 10g). The square wave voltammetry was also performed for the methyl red and carrot juice. The SW voltammogram shown in Fig. 10f indicates a significant difference in current response when exposed to carrot juice and methyl red. This indicates that the nanostructured WO_3_ response was selective to the organic compound, therefore, can be utilized for detecting the methyl sample in carrot juice. A difference in current response pattern can be clearly seen in Fig. 10e (inset). Also, a repeatability in the sensor response can be observed. Although, beetroot juice has a variety of chemical compounds such as Betalains, polyphenols, flavonoids, dietary nitrates, and several other compounds including sugar components that can affect the sensor performance and selectivity. However, analysis of each individual compound of the beetroot juice is challenging and needs techniques such as Folin–Ciocalteu method^76^. Nevertheless most of the experiments were performed after appropriate filtration steps. From the results obtained from the electrochemical tests in the present study, it can be argued that the functionalized WO_3_ nanostructured materials is very sensitive to the synthetic dye (rhodamine 6 G and Methyl red) and selectively detect these compound in the real food sample. From the same study, the detection limit of WO_3_ for Rhodamine 6 G and Methyl red in the food was estimated at around 0.5 µg/ml and ~2 µg/ml respectively.

In summary, the nanorods of WO_3_ prepared using electrochemical etching method, exhibit the areal and gravimetric capacitance of ~225 mF/cm^2^ and ~200 F/g respectively. The study described the simple synthesis method for the synthesis of the nanorod type morphology of tungsten oxide and their modifications. A secondary thin layer of CdS, tellurium, and tungsten selenide was also grown, respectively, in order to improve the robustness and stability of the sensors. The layering causes reduced capacitance values that can be attributed to the charge masking effect due to the layer on WO_3_. However, in some cases, the secondary layer causes impregnation and enhanced anchoring of oriented WO_3_.

Initial studies on the electrochemical detection of three different organic chemicals have demonstrated the sensing ability of the nanostructured tungsten oxide for the various organic compounds that are used in food processing. A systematic change in the current response was observed when the methyl red and β carotene concentration was varied in a buffer solution. The Nyquist plot study showed that systematic conductivity change with the small change in the concentration of rhodamine 6 G in the buffer solution. The present study demonstrated that the application of surface modified nanostructured WO_3_ in electrochemical detection of the dye chemicals such as Rhodamine 6 G and methyl red. Hence, the outcomes from the present study provide an opportunity for the further research in developing a low-cost miniature sensor with a reliable sensing system for commercial food safety use.

## Methods

### Growth of WO_x_ layer

Two distinct approaches were followed in order to grow WO_x_. In the first approach, a spin casting method was used. In this approach, the commercial W-coated Si substrate was first cleaned using ethanol and acetone solution and dried. Next, KOH solution in water (concentration ~0.1 M) was spin cast using a spin coater with a rotation speed of 2500 rotation/min for 30 s. The substrate was initially dried in air and subsequently annealed in a tube furnace for ~2 hours at 650 °C. The authors also conducted the experiments at other temperatures (i.e. 400 °C, 500 °C, 600 °C and 700 °C) in order to examine the effect of temperature on tube morphology. After completion of annealing, the substrate was taken out and finally cleaned in ethanol and dried for further use. In the second approach, electrochemical etching of tungsten film at 30 V for approximately 120 s was performed followed by annealing at 650 °C. Prior to electrochemical tests, the phase and purity were confirmed using Raman spectroscopy^77^. The morphology of WO_x_ was examined using scanning electron microscopy. After these sets of validations, coatings of other layers were also applied to evaluate the performance and cyclic stability of the film.

### Coating of CdS layer

The CdS coating was grown on WOx substrate using a chemical bath deposition method. The chemical bath for CdS deposition was prepared using an aqueous solution of 0.03 M cadmium acetate (Cd(CH_3_COO)_2_), 0.06 M thiourea (CH_4_N_2_S) and 1.0 M ammonia acetate (NH_4_CH_3_CO_2_). The substrate area of ~2.5 cm^2^ was immersed in solution vertically for 2 hours followed by air drying. The as-deposited substrate was heated to 300 °C at a rate of 10 C/min in a vacuum furnace followed by annealing at that temperature for 1 hour in a vacuum (10^−2^ torr).

### Coating of WSe_x_ layer

A WSe_x_ layer was deposited on WO_x_ by annealing WO_x_ coated silicon-substrate in a selenium environment. Elemental selenium (>99.99%) powder was heated in a tube furnace at ~500 °C for 2 hours. Prior to an annealing treatment, the tube furnace was flushed with high purity argon gas. Selenium (Se) powder was poured in a quartz boat and subsequently heated to 500 °C at a rate of 10 °C/min. The deposited Se layer was characterized using Raman spectroscopy and X-ray diffraction. The layer morphology was evaluated using scanning electron microscopy. The energy dispersive spectroscopy (EDS) was employed for the composition analysis of the layer.

### Coating of Te layer

In our continued efforts to evaluate properties of other chalcogenides (apart from WSe_x_)-WOx combination, tellurium is one of the obvious candidates. Various techniques of Te thin film growth has been employed previously such as electrochemical deposition, sputter coating, vacuum deposition^78,79^. In the present study, the Tellurium (Te) film on WO_x_ substrate was prepared by thermal evaporation method due to the fact that the thermal deposition method yields high-quality thin films with a very simple deposition procedure. The pure Te material (99.999%) was used as the source of Te for the deposition on the substrate. The substrate was cleaned with ethanol and dried in air. Te was placed in an alumina boat along with the cleaned substrate in a vacuum tube furnace. A vacuum of 1 × 10^−3^ torr was applied, followed by a continuous heating at a rate of 5 °C/min to 150 °C. The temperature of the furnace was kept at 150 °C for 1 hour followed by furnace cooling to room temperature. The as-deposited substrate was taken out from the cooled furnace and dipped in ethanol to clean the surface. The cleaned surface was dried with flowing air. The morphology of the coated sample was determined using a scanning electron microscope.

### Layer characterization

The phase and structural analysis of the films and layered structures were performed using Raman spectroscopy and X-ray diffraction. Raman spectroscopy was carried out using a portable Raman spectrometer (Raman system: make R 3000 QE) with a 785 nm laser excitation at power ~140 mW. The Raman spectroscopy provides wavelength stability with less than 1 cm^−1^ drift for over a 12 h period. XRD was performed using a Rigaku make R 3000 QE X-ray diffractometer with CuKα radiation over the 2θ range of 20–80° with the step size of 0.005° and a scan rate of 2°/min.

The morphology of each deposited layer was studied using a scanning electron microscope. Morphological examination (surface and cross section) of the thin films was carried out using a field emission scanning electron microscope (Hitachi S-4800 SEM) with a tungsten filament based field emission gun at 5 kV accelerating voltage and ~18 μA emission current. The chemical analysis of the deposited layers was carried out with an energy dispersive X-ray spectroscopic analysis (EDS). For such analysis of the film, an Oxford (X-Max) EDAX detector attached to the SEM, was utilized. EDS analysis was carried out at 20 kV accelerating voltage and high probe current with a given magnification. An AZtecEnergy acquisition method and EDS analysis software synchronized with the X-Max detector was used for mapping and spectral analysis.

### Supercapacitor Performance Measurements

To examine the pseudocapacitive behavior, cyclic voltammetry (CV) experiments were performed in a three-electrode cell system with the layer coated silicon substrate as working electrode, a platinum wire as the counter electrode and saturated Ag/AgCl reference electrode. The CV was performed using a Gamry Potentiostat (equipped with Virtual Front Panel Software) in a potential range of −0.1 and 0.9 V in 1 M Na_2_SO_4_ at various scan rates (5 to 80 mV/s). Samples were cleaned in ethanol prior to CV experiments. In our tests, we measured the CV after stabilizing the WO_x_ electrode in the solution for 60 seconds and also the electrode were immersed in the solution throughout the experiment. The aerial (C_a_) and the specific capacitance (C_g_) for the capacitor was calculated based on data obtained from the CV using the equation^8^:

4 $${C}_{a}=2{[As({\rm{\Delta }}{V})]}^{-1}\int I(V)dV$$

and the specific capacitance of the supercapacitors was calculated using the formula^7,80^:

5 $${C}_{g}=2{[ms({\rm{\Delta }}{V})]}^{-1}\int I(V)dV$$

where $\int I(V){dV}$ is the area under the curve of CV scan, A is the area of the electrode, m is the mass of the working electrode used, s is the scan rate and $\triangle V$ is the working potential window. It can be seen from Eqs (4) and (5), that the capacitance depends on parameters m, s, and $\int I(V){dV}$(determined the swept area). As can be seen as the scan rate increases the capacitance will decrease. The increase in the $\int I(V){dV}$ will increase the capacitance of material. The response from $\int I(V){dV}$ directly depends on the inherent materials and electronic properties of the materials. Ideal supercapacitors should have maximum swept areas.

### Detection of dye

The electrochemical tests were performed using a Gamry PCI4/750 potentiostat equipped with virtual panel software. For the electrochemical tests, a phosphate buffer baseline solution (no additional chemicals) as well as with different concentration of chemicals (Rhodamine 6 G, Methyl orange, and beta carotene) in an electrochemical cell mounted in a quartz cuvette connected to the potentiostat was utilized.

### Detection of dye in the real food sample

For the dye detection in a real food sample, the food sample was chosen based on the fact that the food source should have the similar color (or appearance) to that of our model dye. Based on that, we have selected beetroot juice and carrot juice representative (in color) of Rhodamine 6 G and methyl red, respectively. The beetroot and carrot were purchased from the local market and their respective juices were obtained using a standard juice maker. The juice was filtered using Whatman filter paper. The electrochemical tests (cyclic voltammetry and square wave voltammetry: amplitude: 1.0 V; DC offset: −200 mV; signal frequency: 1.0 Hz) were performed following the same technique mentioned above on the raw juice (baseline) as well as different concentration of chemicals (Rhodamine 6 G and Methyl red) in the respective food (juice). For these tests, the tungsten oxide nanorods layer surface was modified (sensitized) using methyl thiophene aldehyde in order to increase the selectivity for the model chemicals in the juice. For the surface modification, the nanostructured WO_x_ was first dipped into the 1, 2 ethanedithlol overnight followed by dipping of the sample in methyl thiophene aldehyde. Note that thiophene aldehyde based compounds have been earlier used for sensing applications^81^. A schematic diagram showing the surface modification of nanostructured WO_x_ is presented in Fig. 9. To confirm the methyl thiophene aldehyde coating formation on the nanostructured tungsten oxide, the Raman spectroscopy^82^ was carried out by using a 785 nm laser with an excitation power of ~140 mW. The Raman spectrometer provides wavelength stability of less than 1 cm^−1^ drift for over a 12 hour period. The obtained Raman spectrum is shown in Fig. 9b, a distinct aldehyde peak can be seen at wavenumber ~1740 cm^−1^.

### Data availability

The data that support the findings of this study are available from the authors and the same will be provided upon reasonable request.

## Electronic supplementary material

Supplementary Figures

## References

[1] Geim AK, Novoselov KS (2007). The rise of graphene. Nat. Mater..

[2] Chen D, Tang L, Li J (2010). Graphene-based materials in electrochemistry. Chem. Soc. Rev..

[3] Veeramani V, Dinesh B, Chen S-M, Saraswathi R (2016). Electrochemical synthesis of Au–MnO 2 on electrophoretically prepared graphene nanocomposite for high performance supercapacitor and biosensor applications. J. Mater. Chem. A.

[4] Wang G, Zhang L, Zhang J (2012). A review of electrode materials for electrochemical supercapacitors. Chem. Soc. Rev..

[5] Simon P, Gogotsi Y (2008). Materials for electrochemical capacitors. Nat. Mater..

[6] Yoon S, Kang E, Kim JK, Lee CW, Lee J (2011). Development of high-performance supercapacitor electrodes using novel ordered mesoporous tungsten oxide materials with high electrical conductivity. Chem. Commun..

[7] Ratha S, Rout CS (2013). Supercapacitor Electrodes Based on Layered Tungsten Disulfide-Reduced Graphene Oxide Hybrids Synthesized by a Facile Hydrothermal Method. ACS Appl. Mater. Interfaces.

[8] Choudhary N (2016). High-Performance One-Body Core/Shell Nanowire Supercapacitor Enabled by Conformal Growth of Capacitive 2D WS 2 Layers. ACS Nano.

[9] Ghasempour F, Azimirad R, Amini A, Akhavan O (2015). Visible light photoinactivation of bacteria by tungsten oxide nanostructures formed on a tungsten foil. Appl. Surf. Sci..

[10] Kukkola J (2011). Gas sensors based on anodic tungsten oxide. Sensors Actuators B Chem..

[11] Zappa D (2014). Tungsten Oxide Nanowires Chemical Sensors. Procedia Eng..

[12] Drensler S, Walkner S, Mardare CC, Hassel AW (2014). On the pH-sensing properties of differently prepared tungsten oxide films. Phys. status solidi.

[13] Yu Q, Yang X, Chen Y (2016). Electrochemical Detection of Codeine in Pharmaceutical Tablets Using a Tungsten Oxide Nanoparticles and Carbon Nanotubes Modified Electrode. Int. J. Electrochem. Sci..

[14] Righettoni M, Tricoli A, Pratsinis SE (2010). Si:WO3 Sensors for Highly Selective Detection of Acetone for Easy Diagnosis of Diabetes by Breath Analysis. Anal. Chem..

[15] Bhattacharyya D, Smith YR, Misra M, Mohanty SK (2015). Electrochemical detection of methyl nicotinate biomarker using functionalized anodized titania nanotube arrays. Mater. Res. Express.

[16] Kumar P, Mohanty SK, Guruswamy G, Smith YR, Misra M (2017). Detection of Food Decay Products using Functionalized One-Dimensional Titania Nanotubular Arrays. IEEE Sensors Lett..

[17] Li X, Bai J, Liu Q, Li J, Zhou B (2014). WO3/W Nanopores Sensor for Chemical Oxygen Demand (COD) Determination under Visible Light. Sensors.

[18] Kim YS (2005). Room-temperature semiconductor gas sensor based on nonstoichiometric tungsten oxide nanorod film. Appl. Phys. Lett..

[19] Ponzoni A (2006). Ultrasensitive and highly selective gas sensors using three-dimensional tungsten oxide nanowire networks. Appl. Phys. Lett..

[20] Maekawa T, Tamaki J, Miura N, Yamazoe N (1992). Gold-Loaded Tungsten Oxide Sensor for Detection of Ammonia in Air. Chem. Lett..

[21] Solis J, Saukko S, Kish L, Granqvist C, Lantto V (2001). Semiconductor gas sensors based on nanostructured tungsten oxide. Thin Solid Films.

[22] Sekimoto S (2000). A fiber-optic evanescent-wave hydrogen gas sensor using palladium-supported tungsten oxide. Sensors Actuators B Chem..

[23] Wang S-H, Chou T-C, Liu C-C (2003). Nano-crystalline tungsten oxide NO2 sensor. Sensors Actuators B Chem..

[24] Soylak M, Unsal YE, Yilmaz E, Tuzen M (2011). Determination of rhodamine B in soft drink, waste water and lipstick samples after solid phase extraction. Food Chem. Toxicol..

[25] Sun J, Gan T, Li Y, Shi Z, Liu Y (2014). Rapid and sensitive strategy for Rhodamine B detection using a novel electrochemical platform based on core–shell structured Cu@carbon sphere nanohybrid. J. Electroanal. Chem..

[26] Yu L, Mao Y, Qu L (2013). Simple Voltammetric Determination of Rhodamine B by Using the Glassy Carbon Electrode in Fruit Juice and Preserved Fruit. Food Anal. Methods.

[27] Yi Y, Sun H, Zhu G, Zhang Z, Wu X (2015). Sensitive electrochemical determination of rhodamine B based on cyclodextrin-functionalized nanogold/hollow carbon nanospheres. Anal. Methods.

[28] Uslu B, Ozkan SA (2007). Electroanalytical Application of Carbon Based Electrodes to the Pharmaceuticals. Anal. Lett..

[29] Ishihara H (2011). A novel tungsten trioxide (WO3)/ITO porous nanocomposite for enhanced photo-catalytic water splitting. Phys. Chem. Chem. Phys..

[30] Sahin H (2013). Anomalous Raman spectra and thickness-dependent electronic properties of WSe2. Phys. Rev. B.

[31] Zhao W (2013). Lattice dynamics in mono- and few-layer sheets of WS2 and WSe2. Nanoscale.

[32] Okuyama K, Chiba M, Kumagai Y (1979). Epitaxial and Amorphous-Crystalline Phase Transition Growth of Evaporated Te Films. Jpn. J. Appl. Phys..

[33] Boulova M, Lucazeau G (2002). Crystallite Nanosize Effect on the Structural Transitions of WO3 Studied by Raman Spectroscopy. J. Solid State Chem..

[34] Filho AGS (2001). Phase transition in WO3 microcrystals obtained by sintering process. J. Raman Spectrosc..

[35] Baserga A (2007). Nanostructured tungsten oxide with controlled properties: Synthesis and Raman characterization. Thin Solid Films.

[36] Huang J (2015). Large-area synthesis of monolayer WSe 2 on a SiO2/Si substrate and its device applications. Nanoscale.

[37] Sarswat, P. K., Bhattacharyya, D., Free, M. L. & Misra, M. Augmented Z scheme blueprint for efficient solar water splitting system using quaternary chalcogenide absorber material. *Phys*. *Chem*. *Chem*. *Phys*. **18** (2016). 10.1039/c5cp06807j 26762553

[38] Xu J (2015). Tungsten Oxide Nanofibers Self-assembled Mesoscopic Microspheres as High-performance Electrodes for Supercapacitor. Electrochim. Acta.

[39] Zhu M, Meng W, Huang Y, Huang Y, Zhi C (2014). Proton-Insertion-Enhanced Pseudocapacitance Based on the Assembly Structure of Tungsten Oxide. ACS Appl. Mater. Interfaces.

[40] Chen Z (2015). Hierarchical Nanostructured WO3 with Biomimetic Proton Channels and Mixed Ionic-Electronic Conductivity for Electrochemical Energy Storage. Nano Lett..

[41] Qiu M (2016). WO3 nanoflowers with excellent pseudo-capacitive performance and the capacitance contribution analysis. J. Mater. Chem. A.

[42] Yang P (2015). Quantitative Analysis of Charge Storage Process of Tungsten Oxide that Combines Pseudocapacitive and Electrochromic Properties. J. Phys. Chem. C.

[43] Jo C (2013). Block-Copolymer-Assisted One-Pot Synthesis of Ordered Mesoporous WO3− x /Carbon Nanocomposites as High-Rate-Performance Electrodes for Pseudocapacitors. Adv. Funct. Mater..

[44] Tian Y (2014). Synergy of W 18 O 49 and Polyaniline for Smart Supercapacitor Electrode Integrated with Energy Level Indicating Functionality. Nano Lett..

[45] Pal S, Chattopadhyay KK (2016). Tungsten Oxide Nanostructures For Energy Storage And Field Emission Applications. Int. J. Res. Eng. Technol..

[46] Gogotsi Y, Simon P (2011). True Performance Metrics in Electrochemical Energy Storage. Science (80−.)..

[47] Akiyama M, Tamaki J, Miura N, Yamazoe N (1991). Tungsten Oxide-Based Semiconductor Sensor Highly Sensitive to NO and NO 2. Chem. Lett..

[48] Durst RA (1996). Food additive toxicology. Appl. Biochem. Biotechnol..

[49] Gray KM (2016). Illegal Dyes in Food and Spices–A 2006 LGC LC-UV/Visible Method Reviewed and Updated for 19 Dyes. J. Assoc. Public Anal..

[50] Dixit S, Khanna SK, Das M (2013). All India Survey for Analyses of Colors in Sweets and Savories: Exposure Risk in Indian Population. J. Food Sci..

[51] Sachan, D. Toxic industry dyes found in Indian sweets. *Chemistryworld* (2013).

[52] ‘Dangerous dye levels’ found in tikka. *theguardian* (2004).

[53] *U*.*S*. *Food**Imports*. (2015).

[54] *U*.*S*.*-India Bilateral Trade and Investment*. (2016).

[55] Rovina, K., Siddiquee, S. & Shaarani, S. M. Extraction, Analytical and Advanced Methods for Detection of Allura Red AC (E129) in Food and Beverages Products. *Front*. *Microbiol*. **7** (2016). 10.3389/fmicb.2016.00798 PMC4882322 27303385

[56] Rout CS, Govindaraj A, Rao CNR (2006). High-sensitivity hydrocarbon sensors based on tungsten oxide nanowires. J. Mater. Chem..

[57] Keithley RB (2011). Higher Sensitivity Dopamine Measurements with Faster-Scan Cyclic Voltammetry. Anal. Chem..

[58] Chandrashekar BN, Swamy K, Vishnu Mahesh KR, Sherigara BS (2009). Electrochemical Studies of Bromothymol Blue at surfactant Modified Carbon Paste Electrode By using Cyclic Voltammetry. Int. J. Electrochem. Sci..

[59] Mukhopadhyay SC, Gooneratne CP (2007). A Novel Planar-Type Biosensor for Noninvasive Meat Inspection. IEEE Sens. J..

[60] Mohd Syaifudin, A. R., Jayasundera, K. P. & Mukhopadhyay, S. C. A novel planar interdigital sensor based sensing and instrumentation for detection of dangerous contaminated chemical in seafood. In *2009 IEEE Intrumentation and Measurement Technology Conference* 701–706, 10.1109/IMTC.2009.5168540 (IEEE, 2009).

[61] Zia AI (2013). Electrochemical impedance spectroscopy based MEMS sensors for phthalates detection in water and juices. J. Phys. Conf. Ser..

[62] Ide Y (2013). Ternary modified TiO_2_ as a simple and efficient photocatalyst for green organic synthesis. Chem. Commun..

[63] Frederic Cosandey GS, Singhal A (2000). Materials and Processing Issues in Nanostructured Semiconductor GasSensors. JOM-e.

[64] Nava JL, Quiroz MA, Martínez-Huitle CA (2008). Electrochemical treatment of synthetic wastewaters containing alphazurine a dye: Role of electrode material in the colour and COD removal. J. Mex. Chem. Soc..

[65] Panizza M, Cerisola G (2008). Electrochemical Degradation of Methyl Red Using BDD and PbO 2 Anodes. Ind. Eng. Chem. Res..

[66] Gupta AK, Pal A, Sahoo C (2006). Photocatalytic degradation of a mixture of Crystal Violet (Basic Violet 3) and Methyl Red dye in aqueous suspensions using Ag+ doped TiO_2_. Dye. Pigment..

[67] Mascolo G (2007). Photocatalytic degradation of methyl red by TiO2: Comparison of the efficiency of immobilized nanoparticles versus conventional suspended catalyst. J. Hazard. Mater..

[68] Herrmann J-M (2005). Heterogeneous photocatalysis: state of the art and present applications In honor of Pr. R.L. Burwell Jr. (1912–2003), Former Head of Ipatieff Laboratories, Northwestern University, Evanston (Ill). Top. Catal..

[69] Gou Q, Favero LB, Bahamyirou SS, Xia Z, Caminati W (2014). Interactions between Carboxylic Acids and Aldehydes: A Rotational Study of HCOOH–CH2O. J. Phys. Chem. A.

[70] Yajima T, Yu Y, Futamata M (2013). Steric hindrance in cationic and neutral rhodamine 6 G molecules adsorbed on Au nanoparticles. J. Raman Spectrosc..

[71] Choudhuri I, Sadhukhan D, Garg P, Mahata A, Pathak B (2016). Lewis Acid–Base Adducts for Improving the Selectivity and Sensitivity of Graphene Based Gas Sensors. ACS Sensors.

[72] Sathyapalan, A., Sarswat, P. K., Zhu, Y. & Free, M. L. Advanced selective non-invasive ketone body detection sensors based on new ionophores. *Mater*. *Res*. *Express* **1** (2015).

[73] Takahashi S, Watahiki R, Tomida K, Wang B, Anzai J (2013). Voltammetric Studies on Gold Electrodes Coated with Chitosan-Containing Layer-by-Layer Films. Materials (Basel).

[74] Abu Nader PR, Ortiz PI, Mottola HA (1991). Polymer-coated electrode based on the electropolymerization of resole prepolymer mixtures. Anal. Chim. Acta.

[75] Wang Q, Zheng J, Zhang H (2012). A novel formaldehyde sensor containing AgPd alloy nanoparticles electrodeposited on an ionic liquid–chitosan composite film. J. Electroanal. Chem..

[76] Wootton-Beard PC, Brandt K, Fell D, Warner S, Ryan L (2014). Effects of a beetroot juice with high neobetanin content on the early-phase insulin response in healthy volunteers. J. Nutr. Sci..

[77] Sarswat, P. K. & Free, M. L. The effects of dopant impurities on Cu_2_ZnSnS_4_ system Raman properties. *J*. *Mater*. *Sci*. **50** (2016).

[78] Qiu CX, Shih I (1989). Epitaxial growth of tellurium by electrodeposition. Mater. Lett..

[79] Kang J (2011). Reduction of Lattice Thermal Conductivity in Single Bi-Te Core/Shell Nanowires with Rough Interface. Adv. Mater..

[80] Chakravarty D, Late DJ (2015). Microwave and hydrothermal syntheses of WSe 2 micro/nanorods and their application in supercapacitors. RSC Adv..

[81] Li H, Guo J, Zhang X, Chen Z (2012). A Novel Colorimetric and Fluorescent pH Sensor Derived from Iminocoumarin and Thiophene-Carboxaldehyde. Heteroat. Chem..

[82] Sarswat PK, Sathyapalan A, Zhu Y, Free ML (2013). Design, synthesis, and characterization of TPA-thiophene-based amide or imine functionalized molecule for potential optoelectronic devices. J. Theor. Appl. Phys..

