# Supplementary material for: Hybridized Tungsten Oxide Nanostructures for Food Quality Assessment: Fabrication and Performance Evaluation
Source: Sci Rep. 2018 Feb 20;8:3348. doi: 10.1038/s41598-018-21605-5 (PMC5820310; doi:10.1038/s41598-018-21605-5)
Supplement: Supplementary file 1 — Supplementary Figures [file 41598_2018_21605_MOESM1_ESM.docx]

**Hybridized Tungsten Oxide Nanostructures for Food Quality Assessment: Fabrication and Performance Evaluation**

*Pankaj Kumar^1^,* *Prashant K Sarswat^1^*^,*^*, and Michael L. Free^1^*

^1^Department of Metallurgical Engineering, University of Utah, Salt Lake City, UT 84112, USA

**Supporting Information**


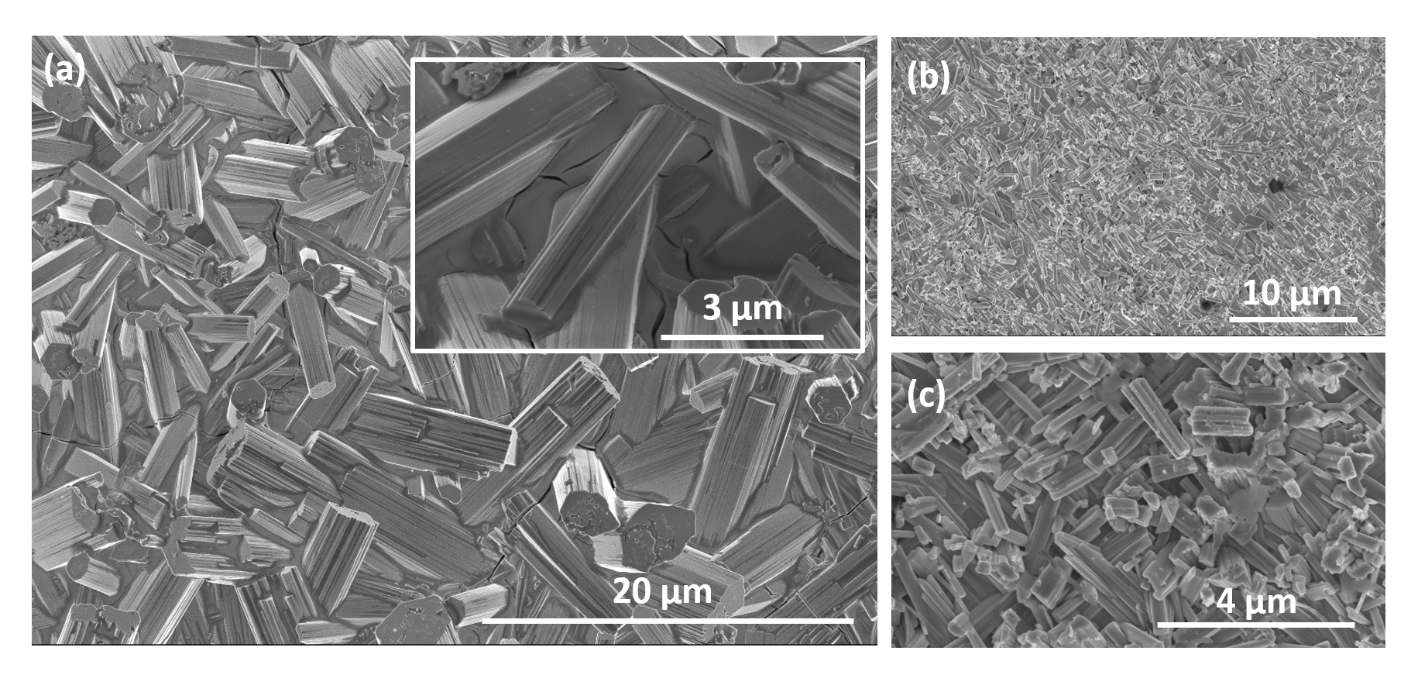


**Figure S1**. Scanning electron micrographs of WOx layer on tungsten coated Si substrate: (a) High magnification SEM image of WOx layer, an inset shows the size and the shape of the rods. (b) SEM image of the WOx coating acquired at lower magnification indicating the distribution of layer on the substrate. (c) SEM micrographs of the WOx acquired at relatively higher magnification to see global variation in the sizes and the shapes of the rods.


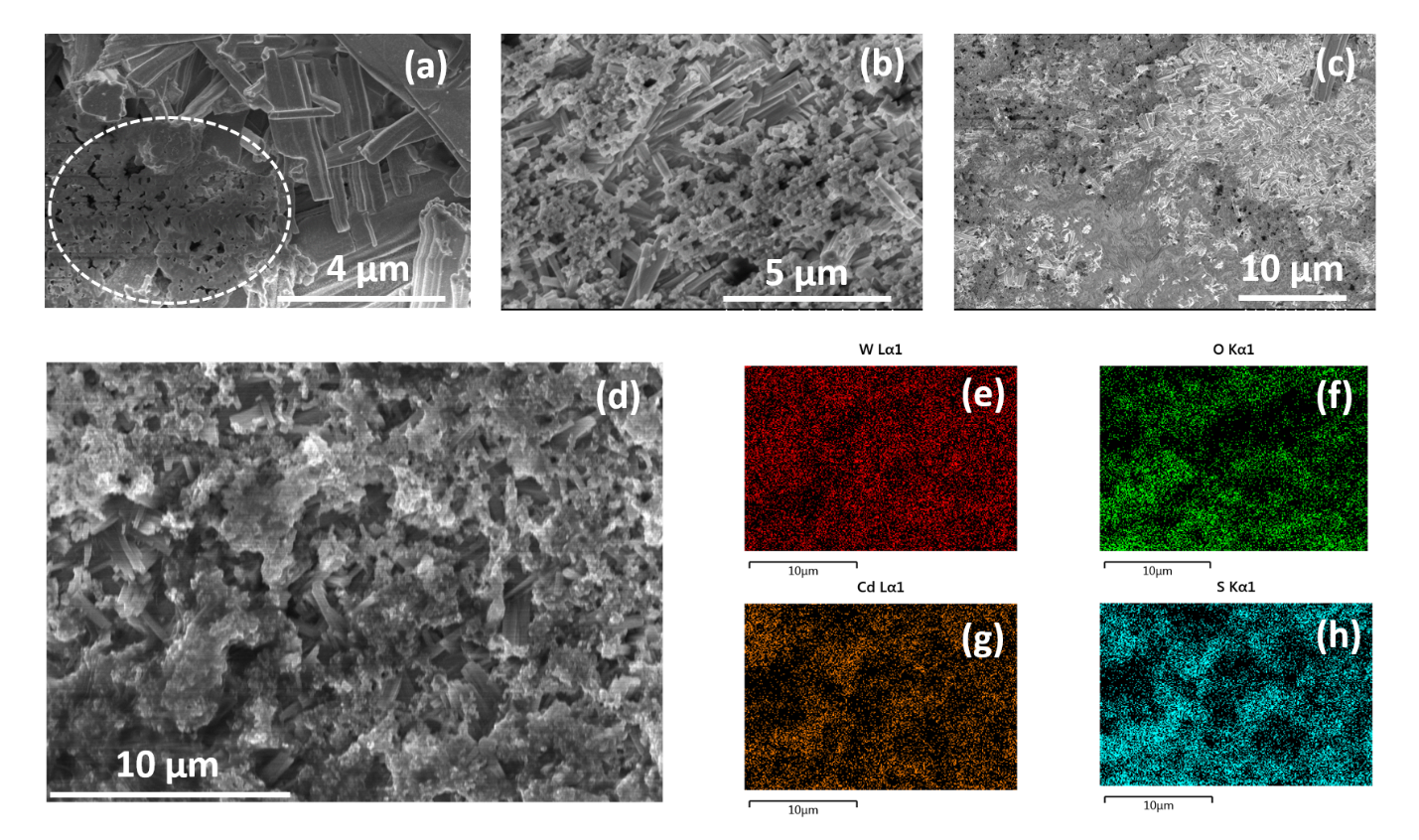


**Figure S2**. Scanning electron micrographs of CdS coating on WOx layer: (a) SEM image of CdS on WOx, circle indicates the CdS layer in the micrographs (b) SEM image acq uired at different magnification showing the coating of CdS layer (c) SEM micrographs acquired at relatively lower magnification to see global coating of CdS (d) SEM image corresponds to the EDS mapping area (e-g) is the elemental EDS mapping of W, O, Cd, S respectively.


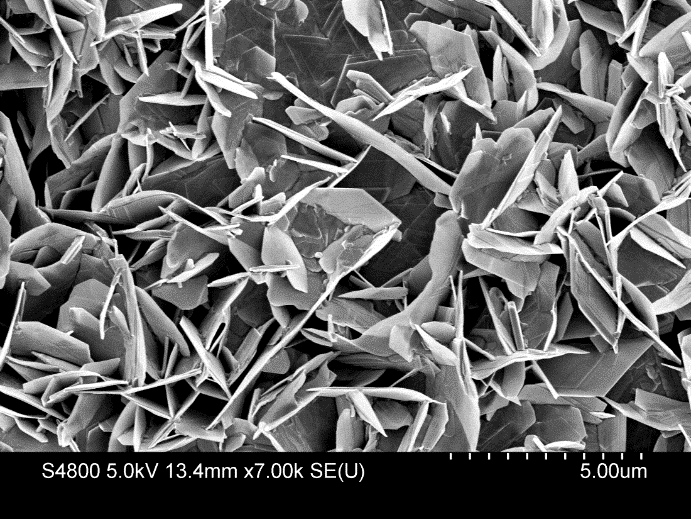


**(b)**


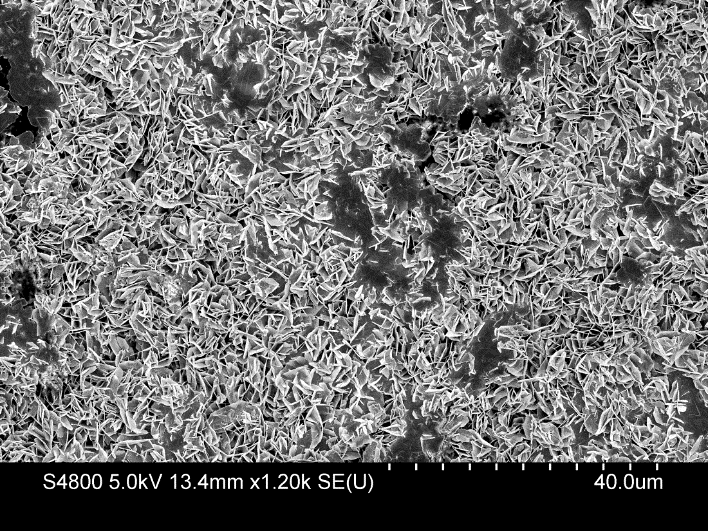


**(a)**


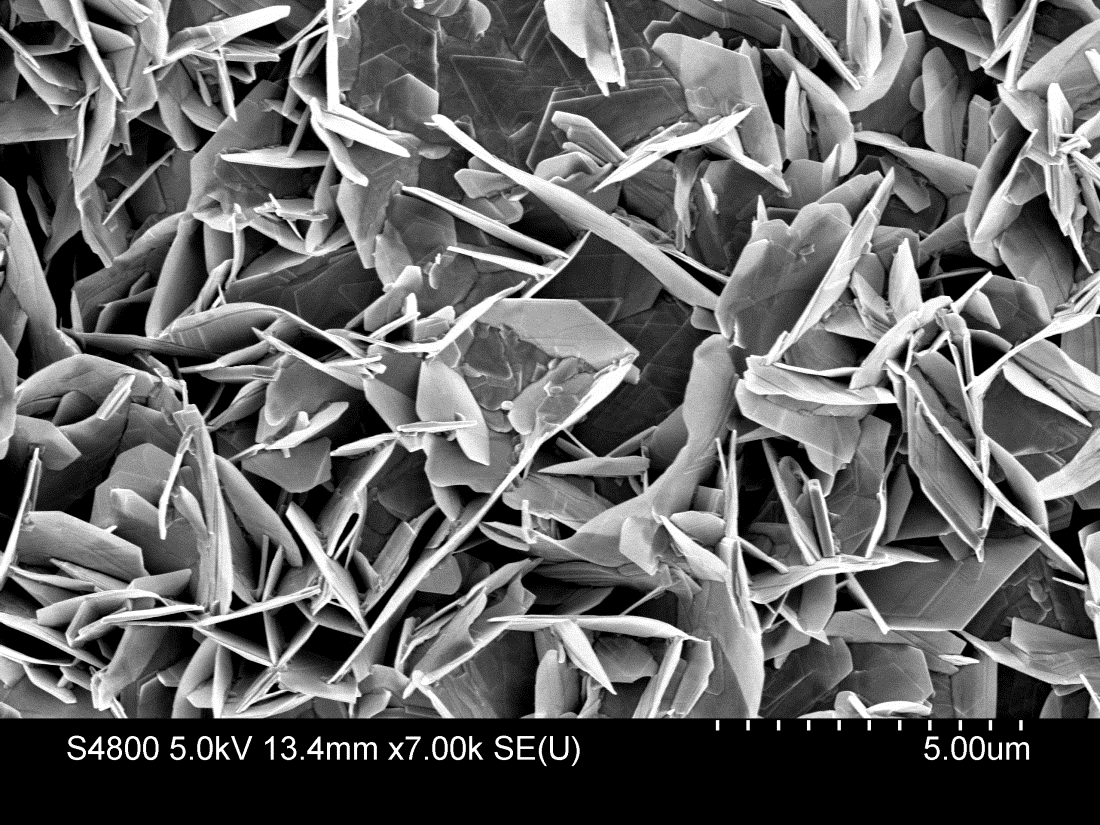


**5 µm**

**(b)**

**Figure S3**. Figure S3. Scanning electron micrographs of WSe2 coating on WOx layer: (a) SEM image taken at relatively lower magnification shows a uniform deposition of WSe2 layer and the flaky morphology (b) SEM image taken at different magnification showing thin flakes (few nm thickness) of WSe2.


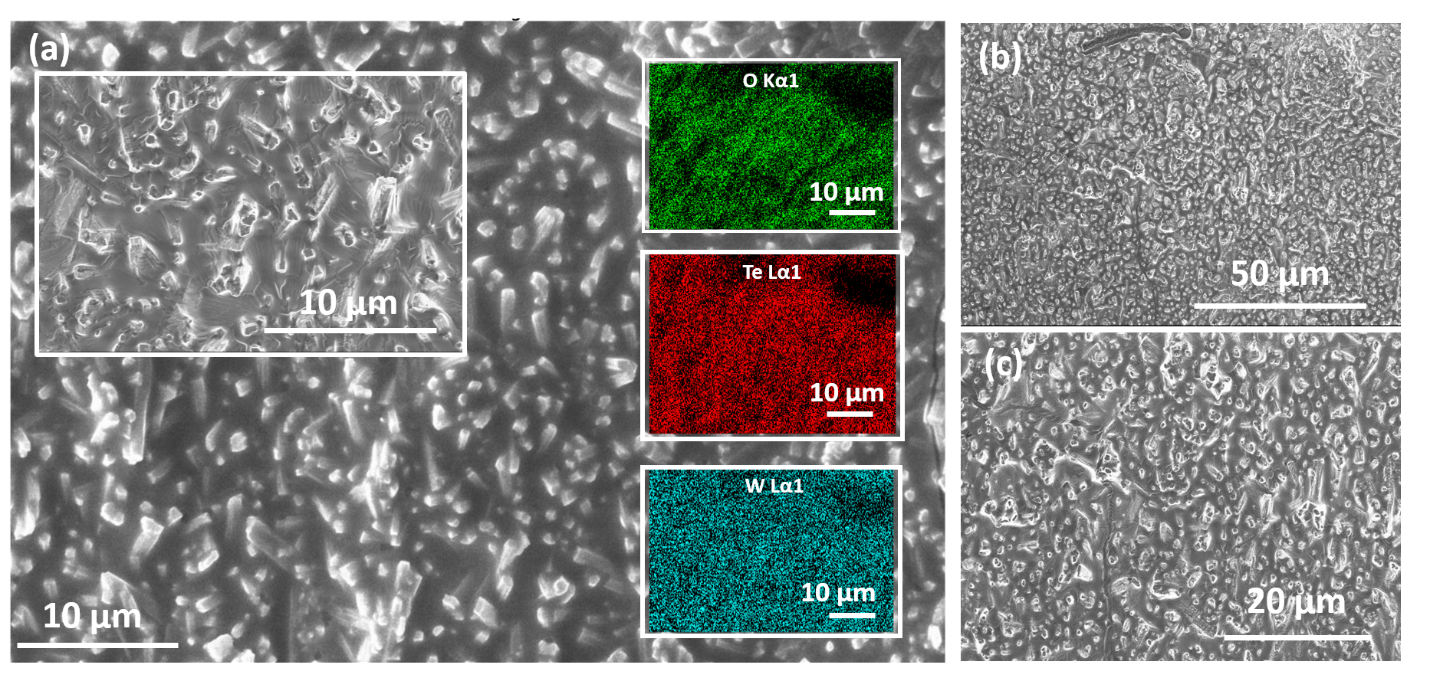


**Figure S4**. Scanning electron micrographs of Te coating on WOx layer: (a) High magnification SEM image of Te later on WOx, insets show the EDS mapping area and corresponding O, Te and W maps (b) SEM image of Te layer acquired at different magnification (c) SEM micrographs acquired at relatively higher magnification to see the morphology of the layer


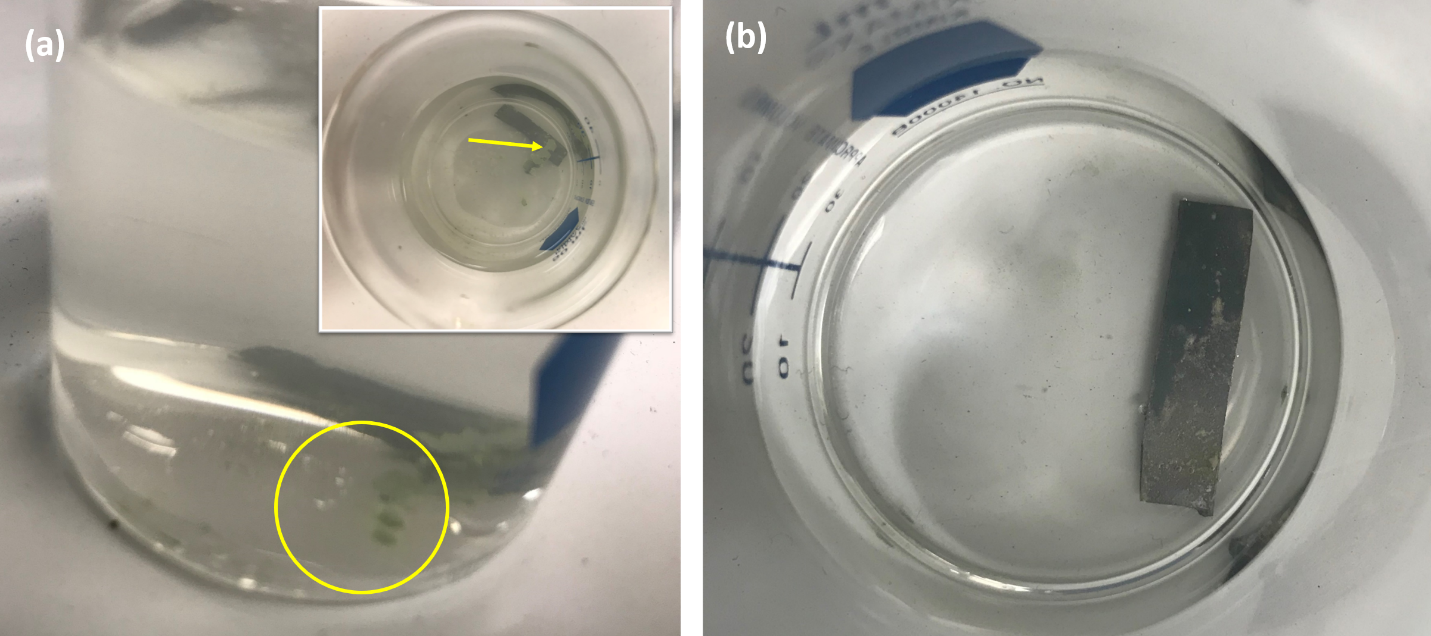


**Figure S5**. The image shows the (a)WO_x_ layer coming off from the Si substrate.The circle indicates the fragments of WO_3_ film from the Si substrate. In inset the arrow indicating the WO_3_ film peeled off from the substrate (b) the CdS coated WO_3_ were intact and no peeling off from the surface, after ultrasonicated for 5 minutes.


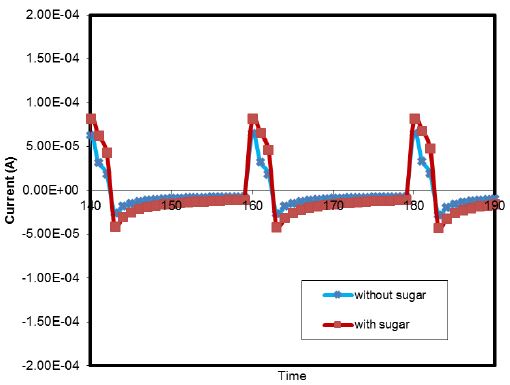


**Figure S6**. Time-current response curve (based in square voltage pulse) for beetroot juice and beetroot juice with added sugar (0.25 gm was added in 1 ml of beet root juice).


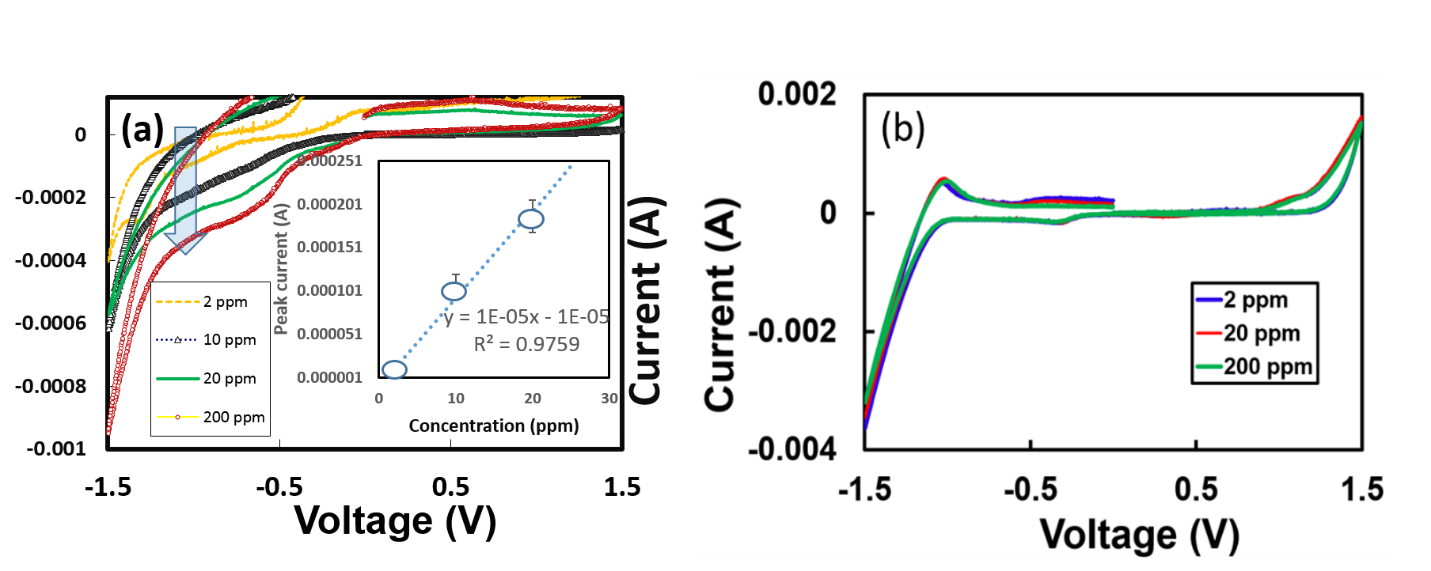


**Figure S7**. The cyclic voltammograms of (a) rhodamine 6G in phosphate buffer (base line) using functionalized nanostructured WO_x_ as electrode showing the change in the electrochemical behavior. Inbox showing a very systematic change in peak current with increase in rhodamine 6G quantity (for the low concentration regime (R^2^ ~ 0.98)). (b) rhodamine 6G in phosphate buffer (base line) using uncoated electrode (bulk and without functionalization) –platinum and reference electrodes combination showing the no change in the electrochemical behavior with increase in the concentration of rhodamine.
